# Structural maturation of the matrix lattice is not required for HIV-1 particle infectivity

**DOI:** 10.1126/sciadv.adv4356

**Published:** 2025-05-09

**Authors:** Long Chen, Yuta Hikichi, Juan S. Rey, Caner Akil, Yanan Zhu, Hana Veler, Yao Shen, Juan R. Perilla, Eric O. Freed, Peijun Zhang

**Affiliations:** ^1^ Division of Structural Biology, Wellcome Centre for Human Genetics, University of Oxford, Oxford OX3 7BN, UK.; ^2^ Virus-Cell Interaction Section, HIV Dynamics and Replication Program, Center for Cancer Research, National Cancer Institute, Frederick, MD 21702-1201, USA.; ^3^ Department of Chemistry and Biochemistry, University of Delaware, Newark, DE 19716, USA.; ^4^ Chinese Academy of Medical Sciences Oxford Institute, University of Oxford, Oxford, UK.; ^5^ Institute for Advanced Study in Physics, Zhejiang University, Hangzhou, Zhejiang 310027, China.; ^6^ Diamond Light Source, Harwell Science and Innovation Campus, Didcot OX11 0DE, UK.

## Abstract

During HIV-1 maturation, the matrix (MA) lattice underlying the viral membrane undergoes a structural rearrangement, and the newly released capsid (CA) protein forms a mature CA. While it is well established that CA formation is essential for particle infectivity, the functional role of MA structural maturation remains unclear. Here, we examine maturation of an MA triple mutant, L20K/E73K/A82T, which, despite replicating similarly to wild-type (WT) in some cell lines, exhibits distinct biochemical behaviors that suggest altered MA-MA interactions. Cryo–electron tomography with subtomogram averaging reveals that, although the MA lattice in immature L20K/E73K/A82T virions closely resembles that of the WT, mature L20K/E73K/A82T virions lack a detectable MA lattice. All-atom molecular dynamics simulations suggest that this absence results from destabilized inter-trimer MA interactions in mature L20K/E73K/A82T mutant virions. These findings suggest that an ordered, membrane-associated mature MA lattice is not essential for HIV-1 infectivity, providing insights into the structural requirements for HIV-1 particle maturation and generation of infectious particles.

## Teaser

CryoET of HIV-1 matrix protein mutants showed that an ordered mature MA lattice is not essential for HIV-1 infectivity.

## INTRODUCTION

The HIV-1 Gag polyprotein precursor, Pr55Gag, includes several domains that serve critical functions during the assembly process: The matrix (MA) domain targets Gag to the plasma membrane and promotes the incorporation of the viral envelope (Env) glycoproteins into virions, the capsid (CA) domain drives Gag oligomerization, the nucleocapsid (NC) domain recruits the RNA genome into assembling particles and promotes particle assembly (*1*), and the p6 domain facilitates the release of newly assembled virions via recruitment of the cellular ESCRT machinery to the site of budding (*2*–*6*). During virus assembly, a primary role of the MA domain is to bring Gag to the inner leaflet (IL) of the plasma membrane via its covalently attached N-terminal myristate group and highly basic region (HBR) (*7*, *8*). The MA HBR promotes Gag membrane binding by engaging in electrostatic interactions with the IL of the plasma membrane (*9*). Phosphatidylinositol 4,5-bisphosphate [PI(4,5)P_2_] is essential for anchoring Gag to the plasma membrane (*10*, *11*) and maintaining its membrane association (*12*). Previous research has demonstrated that PI(4,5)P_2_ directly interacts with HIV-1 MA, inducing a conformational shift that triggers exposure of the myristate (*13*). Furthermore, headgroups of phosphatidylserine (PS), phosphatidylethanolamine, and phosphatidylcholine interact with basic MA residues facing the membrane (*14*). The HIV-1 MA protein forms trimers that assemble into a hexameric lattice with central cavities at points of sixfold symmetry on PS- and PI(4,5)P_2_-enriched membranes (*15*–*17*). During HIV-1 maturation, the MA lattice undergoes structural rearrangement, with mature virions displaying a more regular hexameric lattice and smaller central hexameric apertures compared to their immature counterparts (*18*). Previous studies have shown that the MA domain of HIV-1 Gag is crucial for Env incorporation into virions (*19*–*24*). Mutations that impair MA trimerization block Env incorporation (*23*, *24*) and can be rescued by second-site mutations in MA that restore MA trimer formation, Env incorporation, and particle infectivity (*25*). Notably, many mutations in MA that are defective in Env incorporation cluster around the peripheral tips of the MA trimer near the sixfold cavity (*26*); their compensatory mutations, however, arise at the MA trimer interface (*23*), further implicating the MA trimer as a key structural element in Env incorporation (*23*–*25*). Notably, mutations and deletions in MA that impair Env incorporation can also be effectively overcome by truncating the long gp41 cytoplasmic tail (CT) (*21*, *27*, *28*). Env in immature virions is fusion incompetent. Fusogenicity can be activated by removing the long CT of the transmembrane Env glycoprotein gp41 or upon cleavage of Gag by the viral protease (PR) (*29*, *30*). Gag cleavage by PR induces clustering of Env trimers on the virion that depends on the gp41 CT (*31*). These findings raise the possibility that the structural rearrangement of the MA lattice during virion maturation may modulate Env function, which is critical for successful fusion and entry in the next round of infection.

To understand how the conformational rearrangement of the MA lattice affects viral infectivity, we investigated a previously described revertant of the L20K MA mutant, L20K/E73K/A82T (*32*). Leu^20^ in MA is a highly conserved residue located within the HBR (*33*). The L20K mutation increases Gag association with membrane but reduces HIV-1 infectivity and viral DNA synthesis, relative to the wild type (WT) (*34*). Acquisition by L20K of the two additional mutations, E73K and A82T, rescues L20K defects in virus replication in a cell-type–dependent manner, without reversing the L20K-imposed increase in Gag membrane binding. Intriguingly, unlike the WT and L20K MA, L20K/E73K/A82T MA is resistant to detergent treatment and co-sediments with the core complex (*32*). We previously reported that HIV-1 PR cleaves the CT of murine leukemia virus (MLV) Env when it is incorporated into HIV-1 virions and that the L20K/E73K/A82T mutant exhibits a block in this PR-mediated MLV Env cleavage (*35*). These intriguing features of the L20K/E73K/A82T mutant stimulated further investigations of its MA lattice during maturation within native virions using cryo–electron tomography (cryo-ET), in conjunction with computer simulations and biochemical and virological studies.

## RESULTS

### L20K/E73K/A82T MA forms detergent-resistant oligomers

**Fig. 1.**
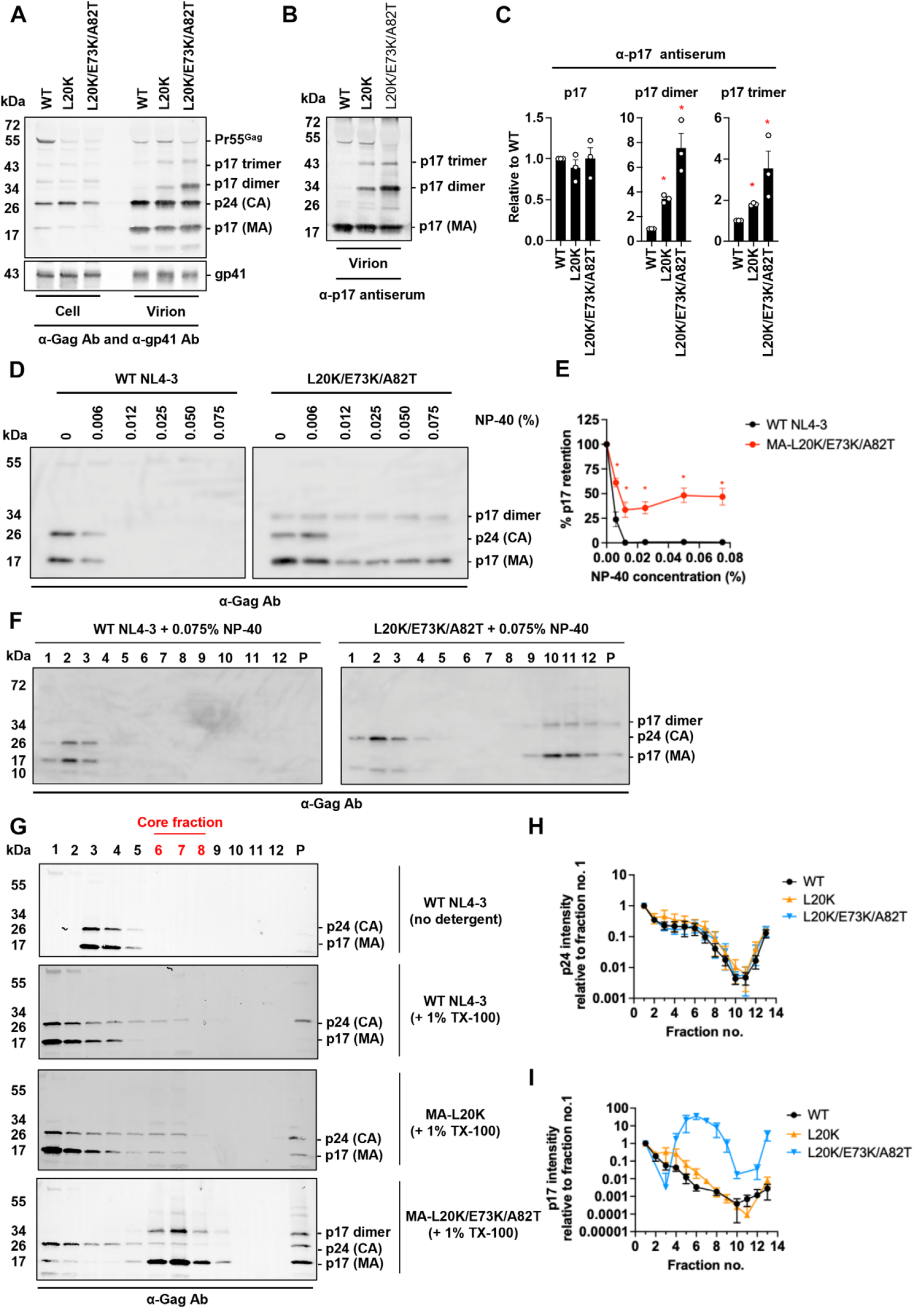
MA-L20K/E73K/A82T exhibits strong MA-MA interactions. (**A** to **C**) Gag processing, Env expression, and incorporation. (A) Cell and virion-associated proteins derived from 293 T cells transfected with WT or MA-mutant pNL4-3 molecular clones were probed with anti-Gag Ab and anti-Env (gp41) antibody (Ab). (B) Virion-associated proteins were probed with anti-p17 (MA) polyclonal Ab. Representative Western blots from three independent experiments are shown. (C) Quantification of p17 monomer/dimer/trimer levels from Western blots in (B). The data are shown as means ± SEM from three independent experiments with statistical significance indicated (*P < 0.05; Student’s *t* test). (**D** and **E**) Membrane stripping assay using the indicated concentration of NP-40. (D) Representative Western blots from three experiments. (E) Quantification of p17 monomer levels from Western blots in (D), indicating the % of p17 retained following detergent treatment relative to the amount present in the absence of detergent. The data are shown as means ± SEM from three independent experiments with statistical significance indicated (*P < 0.05; Student’s *t* test). (**F**) Sedimentation analysis in a 10 to 70% sucrose gradient after stripping the viral membrane with 0.075% NP-40. Representative Western blots from three independent experiments are shown. (**G**) Sedimentation analysis in 3 to 60% OptiPrep gradient after stripping the viral membrane with 1% Triton X-100 (TX-100). Representative Western blots from three independent experiments are shown. The Western blots of the gradient fractions were analyzed using anti-Gag Ab for both the WT and mutant MA. P, pellet fraction. The core fraction highlighted in red was determined by RT assay (fig. S1B). (**H** and **I**) Quantification of p24 (H) and p17 (I) monomer levels from Western blots in (G). The data are shown as means ± SEM from three independent experiments.

Previous studies demonstrated that the L20K mutation, which increases membrane binding of Gag, causes defects in viral replication, single-cycle infectivity, and postentry events compared with WT HIV-1 (*34*, *36*). By propagating the L20K mutant, we identified compensatory second-site mutations in MA (E73K and A82T) that partially rescued replication defects caused by L20K without reversing the increased Gag membrane binding and processing (Fig. 1A) exhibited by L20K (*32*). Western blotting of virion-associated proteins confirmed that the L20K and L20K/E73K/A82T mutations did not affect the amount of p17 (MA), p24 (CA), Pr55Gag, or gp41 (Env) in the virion fraction (Fig. 1A). However, this analysis revealed that both L20K and L20L/E73K/A82T displayed two additional bands, which were less prominent in WT virions, at ~34 and 43 kDa (Fig. 1A). To determine the identity of these additional bands, we probed with anti-p17 antiserum (Fig. 1B) or monoclonal anti-p24 antibody (Ab) (fig. S1A). Consistent with the data in Fig. 1A, L20K and L20K/E73K/A82T showed two bands at 34 and 43 kDa detected by anti-p17 antiserum, but not by monoclonal anti-p24 Ab, suggesting that these bands correspond to a MA dimer and trimer, respectively. The L20K/E73K/A82T mutant exhibits more MA dimer/trimer than L20K (Fig. 1C), suggesting that the compensatory mutations enhance detergent-resistant MA-MA interactions.

To further examine the stability of the L20K/E73K/A82T MA dimer and trimer, we exposed viruses to a range of concentrations of NP-40 and analyzed viral proteins in the pelleted fraction (Fig. 1D). While WT NL4-3 CA and MA were completely solubilized by 0.012% NP-40, the L20K/E73K/A82T MA sedimented in the pelleted fraction in the presence of even higher concentrations of NP-40, with the detergent-resistant MA including both monomer and dimer forms (Fig. 1, D and E). To examine the density profiles of the detergent-resistant MA monomer and multimers, we performed equilibrium sedimentation analysis after exposure of viruses to 0.075% NP-40 (Fig. 1F). Under conditions that disrupt the CA, the mutant MA monomer and multimers preferentially co-sedimented with higher-density fractions, suggestive of the formation of oligomers induced by enhanced MA-MA interactions (Fig. 1F). To extend this finding, we isolated viral cores using a “spin-through” detergent treatment method with 1% Triton X-100 (*37*). While most of WT MA and L20K MA were stripped from the viral core fraction [fractions 6 to 8, determined by reverse transcriptase (RT) assay (fig. S1B)] under these conditions, the MA harboring L20K/E73K/A82T mutations co-sedimented in the core-containing fractions (Fig. 1, G to I), suggesting that the MA oligomer structure maintained its integrity in the presence of the detergent. Sedimentation analysis of mature (fig. S2A) and immature virions (fig. S2B) not treated with detergent revealed that L20K/E73K/A82T enhanced MA-MA interaction in the mature form, with no evidence of increased Gag-Gag interaction in the immature virions. Together, these results suggest a distinct pattern of MA-MA interactions induced by the L20K/E73K/A82T mutations in the context of mature HIV-1 virions. As the L20K/E73K/A82T mutant displays near-WT infectivity and replication kinetics in primary peripheral blood mononuclear cells (PBMCs) and certain T cell lines (e.g., H9 cells) (*32*), these results indicate that the unusual biochemical properties of this mutant do not block particle infectivity. These observations prompted us to carry out structural analysis of the L20K/E73K/A82T mutant.

### Cryo-ET of immature and mature L20K/E73K/A82T VLPs

**Fig. 2.**
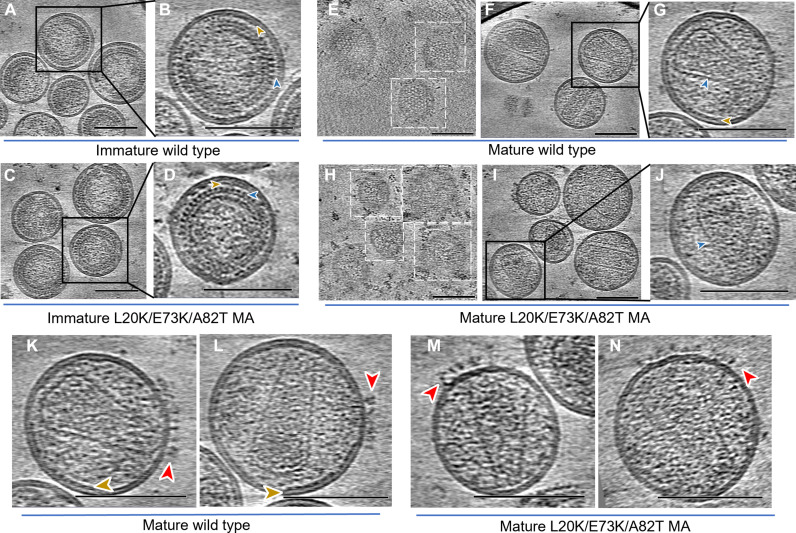
Cryo-ET of immature and mature HIV-1 VLPs of WT and L20K/E73K/A82T MA mutant. (**A** to **D**) Cryo-ET central slices of immature WT [(A) and (B)] and mutant [(C) and (D)] particles, enhanced by summing 10 neighboring slices. The density appears in black with the MA layer and CA layer highlighted by brown and blue arrowheads, respectively. (**E** to **J**) Computational slices through tomographic reconstructions of mature WT [(E) to (G)] and L20K/E73K/A82T mutant [(H) to (J)] particles. [(F), (G), (I) and (J)] Central slices enhanced by summing 10 neighboring slices, displaying side views of conical capsid cores, denoted by blue arrowheads, and MA layers beneath the inner membrane marked by brown arrowheads. The WT shows a regular lattice pattern, while the mutant exhibits disorganized density. Top views [(E) and (H)] focus on slices near the inner membrane surface, showing disrupted MA lattice density in the mutant (H) compared to the regular MA lattice in the WT (E). Gold fiducial markers have been removed from the images for clarity. (**K** to **N**) The Env-containing tomographic slices from mature WT [(K) and (L)] and mutant [(M) and (N)] VLPs. Brown arrowheads indicate the MA lattice, and red arrowheads highlight the Env glycoproteins. Scale bars, 100 nm.

To understand the unusual properties of the L20K/E73K/A82T MA mutant, we imaged immature and mature virus-like particles (VLPs) of this mutant using cryo-ET and compared them with WT VLPs. VLPs were produced from cytomegalovirus (CMV) promoter–driven GagPol expression vectors expressing either WT PR (for mature particles) or a mutant PR containing the inactivating D25N mutation (for immature VLPs), along with Env- and Tat-expressing plasmids. Raw tomograms of immature VLPs displayed a similar morphology for WT and the L20K/E73K/A82T MA mutant, showing striated CA and MA layers (Fig. 2, A to D, blue and brown arrowheads, respectively), indicating lattice formation. In mature VLPs, while conical CA cores are apparent for both WT and the L20K/E73K/A82T mutant (Fig. 2, G and J, blue arrowheads), the mutant MA layers look different from the WT. The mature WT VLPs display a regular, striated MA lattice just below the IL of the lipid bilayer (Fig. 2, F and G, brown arrowhead) and clear lattice arrays in tomographic slices sectioning along the membrane (Fig. 2E), as previously reported (*18*). These features were absent in the mature L20K/E73K/A82T VLPs. No MA lattice was evident underneath the IL of the membrane in mature L20K/E73K/A82T VLPs (Fig. 2, H to J). We observed clustering of Env trimers on the surface of both WT and L20K/E73K/A82T mature VLPs, consistent with the previous fluorescence nanoscopic observations (Fig. 2, K to N) (*31*).

### Structures of immature L20K/E73K/A82T MA trimer and CA hexamer

**Fig. 3.**
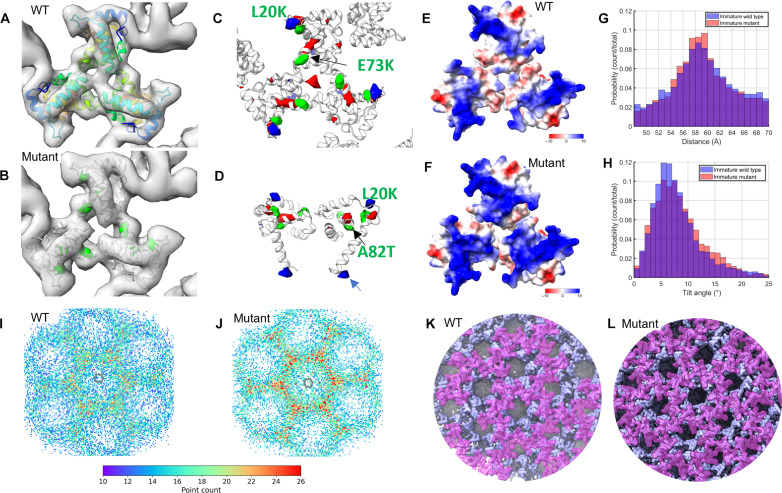
Comparison of immature WT and L20K/E73K/A82T MA mutant. (**A** and **B**) Subtomogram averaging maps of the MA trimer for WT (A) and mutant (B) are shown as gray isosurfaces, viewed from the top toward the virus center. The 7OVQ molecular model (*18*) was fitted into both maps as a rigid body. In the WT, the model is colored from blue (N terminus) to red (C terminus), while, in the mutant, the mutations are highlighted in green. (**C** and **D**) The differential density maps, calculated as mutant minus WT, reveal statistically significant changes at a 1% false discovery rate threshold. The green density marks the locations of mutations. Red and blue, respectively, indicate regions of disappearing and appearing density. (**E** and **F**) Surface electrostatic potential of the MA trimer, derived from Alphafold2-multimer predictions in Colab (*109*, *110*) and MD simulations, is displayed for the WT (E) and mutant (f). Red and blue represent negatively and positively charged areas, respectively. (**G** and **H**) Comparative analysis of trimer-trimer pairings in tomographic reconstructions between the WT (blue) and mutant (orange). The MA-MA distance for WT averaged 59.3 Å with a SD of 5.2 Å, while the mutant recorded 59.2 Å with a SD of 5.0 Å. Tilt angles for the WT were 7.6° (SD 3.9°) versus the mutant’s 8.1° (SD 4.2°). (**I** to **L**) Radial registration analysis between the MA trimer and CA hexamer after mapping back the refined positions and orientations. The MA trimers (*n* = 8000 for both WT and mutant) were projected onto the CA layer to identify intersection points. A heatmap of these intersection points relative to the CA hexamer shows a denser concentration of MA-CA registration in the mutant (J) compared to the WT (I). Representative reconstruction maps illustrate the spatial positioning of MA-CA interactions for the WT (K) and mutant (L).

We further carried out subtomogram averaging of immature MA and CA layers using emClarity and Relion software packages (fig. S3) (*38*, *39*) and obtained immature MA trimer maps for both WT and the L20K/E73K/A82T mutant at 8.0- and 8.3-Å resolution and CA hexamer maps at 4.9- and 5.2-Å resolution, respectively (Fig. 3, table S1, and fig. S4). The MA trimer maps were fitted with the molecular model 7OVQ (*18*) by rigid-body fitting. Comparison of the WT and L20K/E73K/A82T mutant maps revealed a difference at the trimer center, where the central pore of the MA trimer is filled in the WT but open in the mutant (Fig. 3, A and B, and fig. S4, C and D, red arrowhead). To evaluate whether the observed difference is statistically significant, we calculated the difference map between the mutant and the WT MA trimers thresholded at 1% false discovery rate, where density gain and loss are colored in blue and red, respectively (Fig. 3, C and D). The difference map displayed pairs of density “gain” and “loss,” which are largely associated with the mutations (Fig. 3, C and D; L20K and A82T). In addition to the significant density loss in the center of the L20K/E73K/A82T MA trimer, there was an unpaired density gain at the C terminus of L20K/E73K/A82T MA pointing to the CA layer (Fig. 3D, blue arrow), suggesting that this region is likely less flexible than the WT counterpart. Additionally, the electrostatic potential on the surface of MA facing the membrane is also more positive than the WT, due to the L/K and E/K changes in the L20K/E73K/A82T mutant (Fig. 3, E and F). Further comparative analysis on the spatial arrangement of MA trimers and the underlying CA hexamers, namely, the distance and angle between the nearest neighbors, showed a consistent profile between WT and L20K/E73K/A82T (Fig. 3, G and H, and fig. S4, G and H), suggesting that the L20K/E73K/A82T mutations have a minimal effect on the structural organization of the immature MA and CA lattices.

### The L20K/E73K/A82T mutations enhance immature MA and CA lattice registration

In immature Gag, the MA domain, which is anchored to the membrane and forms a trimeric lattice, is flexibly linked to the CA domain, which forms a lattice of hexamers below the MA layer. We hypothesize that the MA lattice might be in register with the CA lattice. To visualize both the MA trimer lattice and the CA hexamer lattice, we placed MA trimer and CA hexamer sub-volumes back in the original tomograms (fig. S5, A and B). Subsequent analysis of MA trimer localization from WT and L20K/E73K/A82T mutant VLPs showed that the MA lattices are very similar but are less well ordered than the CA lattice (fig. S5, C to F).

We next investigated whether the linker between MA and CA constrains the relative position of these two domains and whether the MA and CA lattices are in register. To understand the relationship between MA and CA lattices, we projected the center of MA trimers radially onto the CA lattice layer and plotted the distribution of intersection points with reference to the center of the immature CA hexamer (Fig. 3, I to L). While the overall distribution is relatively dispersed, there is a slight preference for the MA trimer to be in register with the CA trimer (or threefold axis) for WT VLPs (Fig. 3, I and K). But more intriguingly, in L20K/E73K/A82T mutant VLPs, the intersection points became concentrated at the threefold axis, and the registration between the MA trimer and the CA trimer is much stronger than WT (Fig. 3, J and L). This is consistent with the observation of a more ordered MA C terminus in the L20K/E73K/A82T mutant MA compared to the WT (Fig. 3D). The L20K/E73K/A82T mutations induce changes in the spatial organization of Gag, which may be attributed to the observed increased Gag processing in the L20K/E73K/A82T mutant (Fig. 1A).

### MD simulations of immature MA trimer-lipid interactions

**Fig. 4.**
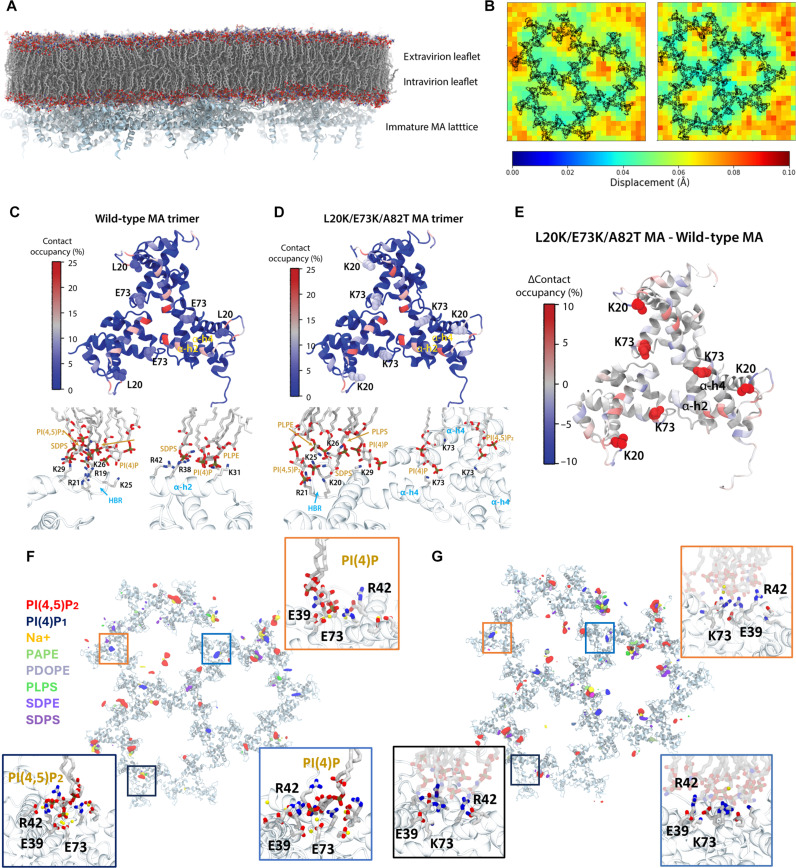
Lipid interactions with the immature MA lattice from molecular dynamic simulations. (**A**) Model of the immature WT MA lattice embedded in an asymmetric lipid membrane of native HIV-1 lipidomics composition. (**B**) Time average map of lateral lipid displacement over 1 μs for the membrane in complex with immature WT MA lattice (left) or the immature L20K/E73K/A82T MA lattice (right). The average position of MA is colored in black. (**C**) Top: WT MA trimer colored by lipid contact occupancy. Bottom: Representative protein-lipid interactions of positively charged residues (black) in the HBR and helix 2 with intravirion leaflet lipids (tan). (**D**) Top: L20K/E73K/A82T MA trimer colored by lipid contact occupancy. Bottom: Interaction of residues at the HBR and E73K from helix 4 with intravirion leaflet lipids. (**E**) L20K/E73K/A82T MA trimer, colored by the difference in lipid contact occupancy with respect to WT MA. (**F** and **G**) Per-lipid occupancy maps for the intravirion leaflet lipid headgroups in the presence of (F) WT MA or (G) L20K/E73K/A82T MA. Insets show specific interactions between PI(4,5)P2 and PI(4)P lipids in the membrane, with E39 and E73 (Na + ion coordination) and R42 (salt-bridge) at the WT MA trimer centers while lipid-residue interaction at L20K/E73K/A82T MA trimer centers coordinated by R42 are nonspecific. Lipid abbreviations for non-PIP lipids follow the CHARMM nomenclature: PAPE, 1-palmitoyl-2-arachidonyl-phosphatidylethanolamine; PDOPE, 1-palmitoyl-2-docosahexaenoyl-phosphatidylethanolamine; SDPE, 1-stearoyl-2-docosahexaenoyl-phosphatidylethanolamine; 1-PLPS, palmitoyl-2-linoleoyl-phosphatidylserine; SDPS, 1-stearoyl-2-docosahexaenoyl-phosphatidylserine.

To determine the effect of L20K/E73K/A82T mutations on MA and lipid interactions, molecular dynamics (MD) simulations were used to determine the regulatory effects of immature WT and L20K/E73K/A82T MA mutant proteins on lipid dynamics within the HIV-1 lipid membrane. The MD model encompassed an immature MA lattice [derived from Protein Data Bank (PDB) IDs 7TBP (*40*) and 2LYB (*14*) as indicated in Materials and Methods] embedded into an asymmetric lipid membrane of native HIV-1 lipidome composition (Fig. 4A and table S2). We measured the instantaneous lateral displacement of lipids in the intravirion leaflet over 1-μs trajectories (table S3, simulations 4 to 6) using a vector field approach (*41*). Although the patterns of correlated lipid motion in the IL are transient (nanosecond scale), we observe the formation of static regions in the membrane due to the presence of the MA lattice (fig. S6 and movie S1).

Notably, the average displacement over time indicated that these static regions in the intravirion leaflet correlate with the position of the MA lattice, suggesting that the MA lattice aggregates lipids in the intravirion leaflet (Fig. 4B and fig. S6). We also observe lipid dynamics being regulated by cholesterol, which is highly enriched in the HIV-1 membrane (table S2). MA hexamer centers with fewer cholesterol molecules present higher average lateral lipid displacement (fig. S6, C and D).

To further investigate specific MA regions related to aggregation of lipid headgroups, we conducted a comparative analysis of lipid contact occupancies between WT and mutant MA over the full trajectory. In both WT and L20K/E73K/A82T mutant MA, lipids aggregate with high lipid occupancies near positively charged amino acids, particularly at the HBR due to interactions with R21, K25, K26, and K29, as well as near helix 2 via interactions with R38 and R42 (Fig. 4, C and D). Further comparative analysis revealed significant differences in lipid contact occupancies, with the L20K/E73K mutations increasing average occupancy at binding sites on the HBR and helix 2 and helix 4 (Fig. 4E). This suggests that L20K/E73K MA mutations enhance lipid-protein contact occupancies, consistent with its increased positive electrostatic potential on the lipid binding surface (Fig. 3, E and F).

In addition, to elucidate the differences in the cryo-ET density between WT and mutant MA, we calculated the occupancy of lipid headgroups in the intravirion leaflet. From these calculations, we observed regions of high occupancy near trimer centers in the WT MA that were absent in the occupancy maps for mutant MA. Conversely, mutant MA displayed greater aggregation of lipid headgroups near the HBR and helix 4 (fig. S7).

Further analysis of occupancy maps by lipid species indicated that the density at the trimer centers corresponds to specific binding of PI(4,5)P_2_ and PI(4)P lipid headgroups, while lipid aggregation in other regions appeared nonspecific (Fig. 4, F and G). MD simulation trajectories reveal that at the trimer centers, E39 and E73 aggregate and coordinate Na^+^ ions with the phosphate groups of the PI(4,5)P_2_ and PI(4)P headgroups. This ion coordination, along with salt-bridge interactions with R42, stabilizes PIPs at the trimer centers of the WT MA lattice (Fig. 4F). In the L20K/E73K/A82T mutant MA, however, E73 is substituted with K73, which forms salt-bridges with E39 and E41 and transiently with other lipid headgroups but does not contribute to Na^+^ ion coordination. As a result, interactions at the trimer centers in mutant MA are only mediated by salt bridges with R42, making them less stable compared to WT MA (Fig. 4G and movie S2).

### The L20K/E73K/A82T mutant does not form a WT-like mature MA lattice

**Fig. 5.**
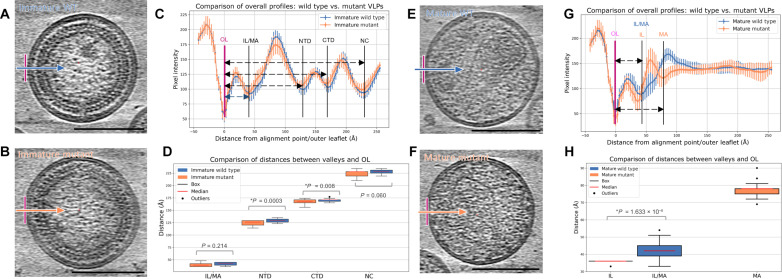
Analysis of mature MA lattice. (**A** to **D**) Radial density profiles and distances of immature WT (blue) and mutant (orange) VLPs, measured from the OL of viral membrane. Profiles are aligned to the OL (the first valley). Representative central slices of immature WT (A) and L20K/E73K/A82T mutant (B) VLPs, enhanced by summing 10 neighboring slices, are demonstrated. Comparison of radial density profiles and distances between valleys and OL are plotted [(C) and (D)], revealing similar profiles and stable Gag protein organization during membrane assembly. Slight differences in the distances from IL/MA, N-terminal domain (NTD), C-terminal domain (CTD), and NC to OL suggest that the mutant Gag shifts slightly toward IL. This indicates a potential increase in membrane binding affinity for the mutant. (**E** to **H**) Radial density profiles and distances of mature WT and L20K/E73K/A82T mutant VLPs, measured from the OL of viral membrane. Representative central slice of mature WT (E) and mutant (F) VLPs, enhanced by summing 10 adjacent slices, is presented. Comparison of radial density profiles and distances between valleys and OL are plotted [(G) and (H)], displaying an additional valley further away from the OL in mutant VLPs. Each analysis includes over 20 complete VLPs per sample, with defocus values ranging from 4 to 5 μm. Scale bars, 100 nm.

As both CA and MA undergo major structural rearrangements upon PR-mediated Gag cleavage, converting from the immature to the mature configuration (*18*, *42*), we next investigated the effect of L20K/E73K/A82T mutations on the structural maturation of VLPs. Using the same strategy used to solve the structure of the MA layer in immature particles (fig. S3), we determined the structure of the mature WT MA trimer at 7.3-Å resolution by subtomogram averaging (fig. S8, A to D, and table S1). The resulting structure overlaps closely with the previously published mature MA trimer structure, as well as the mature MA trimer lattice (fig. S8, E and F) (*18*). In contrast, the mature L20K/E73K/A82T MA did not yield consistent subvolumes through either template matching or template-free approaches. Considering that no apparent MA density was observed at the IL of the viral membrane in raw tomograms of mutant VLPs (Fig. 2, I to K), we attempted to locate the MA density layer using a density profiling approach (fig. S9). By analyzing more than 20 complete VLPs, the radial density distribution profiles were aligned to the density valley of the outer leaflet (OL) and plotted along the radial distance with SDs (fig. S9E). The distance of each density valley from the OL could then be calculated (fig. S9, D to F). To validate this method, we carried out such measurements using immature VLPs from both WT and the L20K/E73K/A82T mutant. Consistent with the tomography analysis, the radial density distribution profile from the immature mutant VLPs matches well to the profile from immature WT VLPs (Fig. 5, A to C). There were no significant differences for the radial positions of MA, NC, and very small changes (~3 Å) for N-terminal domain of CA and C-terminal domain of CA between the WT and the mutant (Fig. 5D). While mature WT VLPs showed the same MA localization as immature WT VLPs (Fig. 5, E to G, IL/MA position), the mature L20K/E73K/A82T mutant VLPs displayed two separate density valleys. The first density valley corresponds to the IL of the membrane, which is located slightly closer (~7 Å) to the OL compared to the IL/MA layer in WT (Fig. 5, G and H). The second density valley, located ~77 Å (SD, 4.7 Å) from the OL, was not present in mature WT VLPs (Fig. 5, G and H, MA). These results suggest that the L20K/E73K/A82T mutant MA may have detached from the viral membrane upon maturation.

These findings prompted us to examine virions produced by the original single MA mutant L20K. The L20K mutation results in impaired endogenous reverse transcriptase activity and reduced viral infectivity (*34*, *36*). We produced and imaged immature and mature L20K mutant VLPs by cryo-ET as described above for the WT and the L20K/E73K/A82T revertant (table S4). Both immature and mature L20K VLPs exhibit a clear MA lattice at the IL of the membrane (fig. S10, A to I, brown arrowheads). Further subtomogram averaging of mature L20K MA lattice resulted in a density map which is indistinguishable from the mature WT MA lattice (fig. S10, J and K). Therefore, the loss of MA lattice appears to be a feature of the mature L20K/E73K/A82T mutant.

### MD simulations of the mature MA trimer lattice

To explore the effect of L20K/E73K/A82T mutations on the mature lattice, we turned to MD simulation. First, an atomistic model for an extended MA lattice was derived for WT MA, L20K MA, and L20K/E73K/A82T MA from the cryo–electron microscopy (cryo-EM)/cryo-ET structures presented herein as described in the Supplementary Materials. To simulate the stability of the mature WT MA and mature mutant MA lattices, we quantified the structural defects in the MA lattice through perturbation MD simulations in which we progressively adsorbed the mature MA lattice into the membrane, effectively varying the chemical environment (table S3, simulations 7 to 9; and movie S3). During simulations, we measured the trimer-trimer distances for every pair of neighboring MA trimers (fig. S11) and quantified lattice structural stability by analyzing the distribution of trimer-trimer distances for MA trimers at the outer edge of the lattice. The latter is due to inner trimers being restrained by their neighbors.

**Fig. 6.**
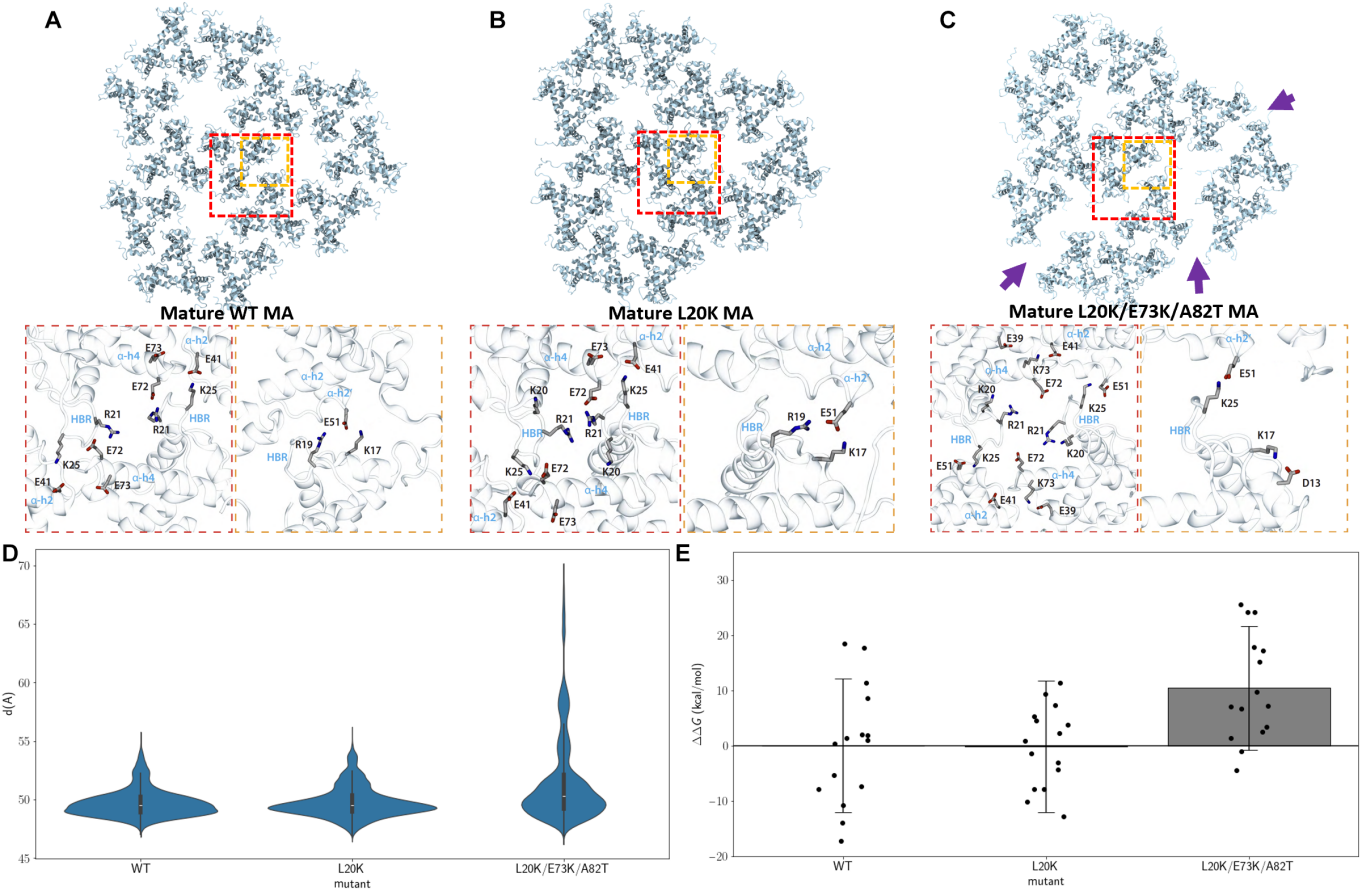
MD simulations of mature MA interfaces. (**A** to **C**) Mature MA lattice and inter-trimer interactions for WT MA (A), L20K MA (B), and L20K/E73K/A82T MA (C). (**D**) MA trimer-trimer distance distributions through lattice perturbation MD simulations. (**E**) Mature MA intertrimer estimated binding affinity compared to WT MA trimer interfaces via MM-GBSA calculations.

Analyzing the trajectories resulting from our MD simulations of the MA lattice structure during the perturbative simulations (table S3, simulations 7 to 9), we observed notable alterations at the trimer-trimer interface in mature lattices due to the L20K/E73K/A82T mutations (Fig. 6, A to C). Specifically, the mature WT MA lattice is typically stabilized by a network of inter-trimer salt bridge interactions involving residues from the HBR and glutamates from helices 4 and 2, namely, K17-E51, R19-E51, R21-E72, and K25-E41 (Fig. 6A). These salt bridges contribute significantly to the structural integrity and stability of the lattice. Although the L20K MA lattice maintains the same inter-trimer interactions as WT MA (Fig. 6B), the L20K/E73K/A82T mutations disrupt these critical inter-trimer salt bridges while promoting intra-trimer interactions, notably K73-E41, K73-E39, R21-E72, and K17-D13, which significantly alter the network of stabilizing forces within the lattice. Additionally, these mutations result in the displacement of the K25-E41 interaction to a new configuration between K25-E51 (Fig. 6C, fig. S12, and movie S4).

Comparing the trimer-trimer distance distributions aggregated over six replicates of the perturbation/adsorption simulations, we observe that the distributions for WT MA and L20K MA lattices are similar according to the Jensen-Shannon distance of 0.07 (*43*, *44*); the Jensen-Shannon distance evaluates the similarity between probability densities with a value between 0 and 1, 0 being identical and 1 most dissimilar. In contrast, the L20K/E73K/A82T MA lattice exhibited breaking events, with inter-trimer distances extending beyond 60 Å. This results in a multimodal distribution that differs significantly from that observed for WT and L20K MA lattices, with a Jensen-Shannon distance of 0.30 (Fig. 6D). Additionally, tracking the occurrence of breaking events through the perturbation simulations revealed that the L20K/E73K/A82T MA presented a broken lattice on average 60% of the time compared to 17% of the time for WT MA and 6% for L20K MA (fig. S11D and movie S5). These results suggest that the WT and L20K mutant form more resilient MA lattices when compared to the L20K/E73K/A82T mutant, consistent with the result from cryo-ET.

Furthermore, we estimated the energetic favorability of the mutations for the self-association of MA trimers by calculating the binding affinity of trimer pairs throughout the simulations using the molecular mechanics generalized born surface area MM-GBSA method (*45*–*47*). While L20K MA shows a negligible difference in binding affinity compared to WT MA trimer interface (ΔΔ*G* = 0.2 kcal/mol), the L20K/E73K/A82T mutations result in a more energetically unfavorable inter-trimer interface, with an average energetic penalty of ΔΔ*G* = Δ*G*_L20K/E73K/A82T_ − Δ*G*_WT_ = 10.4 kcal/mol (Fig. 6E). As a result, we conclude that the L20K/E73K/A82T MA mutant is less likely to form higher-order trimeric assemblies.

These alterations not only compromise the stability of the mature MA lattice but may also explain why the mature L20K/E73K/A82T mutant MA appears to detach from the viral membrane. The enhanced intra-trimer interactions in the mutant further provide an explanation for the dimer and trimer observed for the MA mutant by SDS–polyacrylamide gel electrophoresis (PAGE) (Fig. 1, A to C). The purified WT and L20K/E73K/A82T mutant MA proteins display distinct features. Not only does the purified L20K/E73K/A82T mutant MA exist in monomer, dimer and trimer forms in the SDS-PAGE (fig. S13, A to C), it also forms filamentous structures seen in cryo-EM micrographs, in contrast to the WT MA (fig. S13, D and E).

### Mutational analyses of the mature MA trimer-trimer interface

**Fig. 7.**
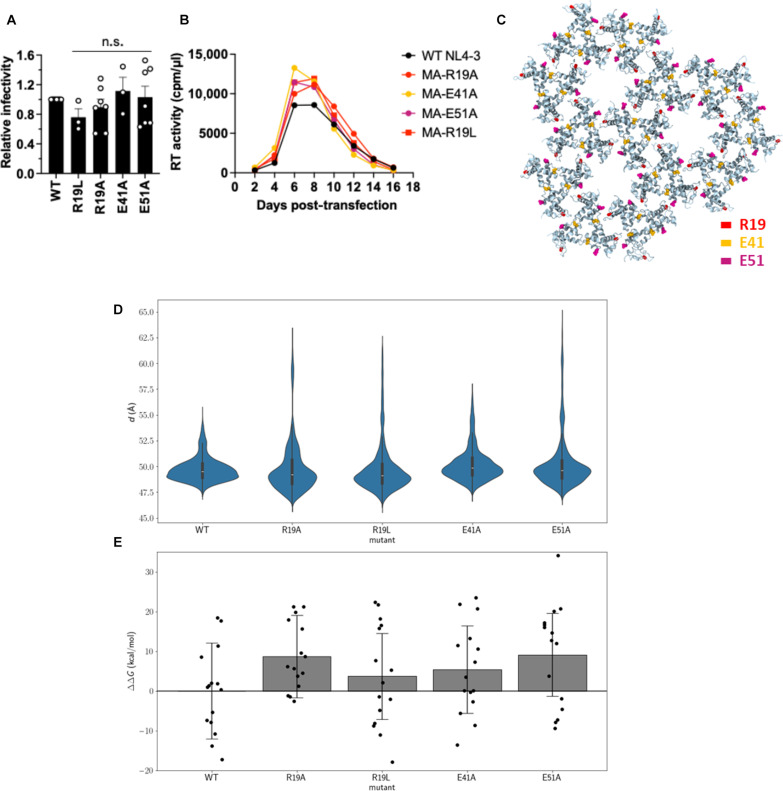
Mutations at the inter-MA trimer interface do not impair HIV-1 infectivity. (**A**) Single-cycle, cell-free viral infectivity of the indicated MA mutants. RT-normalized virus stocks were used to infect TZM-bl cells. Luciferase activity was measured at 48 hours postinfection. Relative infectivity is shown, normalized to 1 for WT NL4-3. Data from at least three independent experiments are shown as means ± SEM. n.s., not significant, one-sample *t* test. (**B**) Replication kinetics of the indicated MA mutants. The H9 T cell line was transfected with WT NL4-3 or the indicated MA-mutant proviral clones. Virus replication kinetics were monitored by measuring RT activity at the indicated time points. Data are representative of three independent experiments. (**C**) Mature MA lattice showcasing the position of the mutations in the MA inter-trimer interfaces. (**D**) MA trimer-trimer distance distributions for each salt-bridge–disrupting mutation R19A, R19L, E41A, and E51A through six replicas of MA lattice perturbation MD simulations. (**E**) Estimated mature MA intertrimer binding affinity compared to WT MA trimer interfaces via MM-GBSA calculations.

Our previous studies demonstrated that MA mutations, such as MA-R19L, do not significantly impair viral replication in T cell lines or monocyte-derived macrophages despite being present at the putative mature MA inter-trimer interface (*48*). This finding supports our results for the L20K/E73K/A82T mutant, suggesting that disruption of the mature MA lattice may not affect virus replication or infectivity. To further explore this question, we introduced alanine mutations at R19, E41, and E51 to disrupt the mature MA trimer-trimer interaction. Single-cycle infectivity and multicycle replication kinetics analysis demonstrated that these alanine mutations do not impair infectivity or viral replication (Fig. 7, A and B).

To assess the effects of these mutations on the mature MA lattice, we performed MD perturbation simulations on the mature MA lattices containing the R19A, R19L, E41A, and E51A mutations (Fig. 7C and table S3, simulations 10 to 13). We observe that the trimer-trimer distance distributions for all these mutants extend beyond the expected range for WT MA, differing from the WT trimer-trimer distance distribution with Jensen-Shannon distances of 0.33, 0.32, 0.18, and 0.24 for the R19A, R19L, E41A, and E51A mutations, respectively. This suggests that the MA trimers separate from one another in lattice-breaking events (Fig. 7D). Moreover, the simulations suggest that the trimer-trimer interfaces in these mutant MA lattices also become energetically unfavorable compared to those of the WT MA lattice, with the average binding affinity incurring penalties of ΔΔ*G* = 8.7, 3.7, 5.4, and 9.1 kcal/mol for R19A, R19L, E41A, and E51A, respectively (Fig. 7E). Although the effects of these mutations on the MA lattice are not as pronounced as those seen in the L20K/E73K/A82T mutant, the salt-bridge–disrupting mutations are predicted to compromise the MA lattice both structurally and energetically.

## DISCUSSION

The HIV-1 MA domain plays a critical role in Gag targeting to the plasma membrane and Env incorporation into virions. Structural studies have shown that MA trimers form a hexagonal lattice on virions, with its organization influenced by PR-mediated Gag cleavage (*18*). However, the biological significance of this structural rearrangement in the MA lattice following Gag cleavage remains unclear. In this study, we compared the structure of the immature and mature MA lattices in WT and L20K/E73K/A82T MA mutant virions using biochemical, structural, and computational approaches. Our results demonstrate that, while the L20K/E73K/A82T MA mutant exhibits distinct biochemical MA properties compared to WT, it maintains similar levels of infectivity in the H9 T cell line and in PBMCs (*32*). Structural and computational analyses further reveal that the L20K/E73K/A82T mutant lacks a well-organized mature MA lattice in virions due to unstable inter-trimer interactions despite forming a WT-like immature MA lattice. At this time, we cannot exclude the possibility that the L20K/E73K/A82T MA mutant forms a mature lattice that is too disordered to be detected by STA. Additionally, mutations in MA residues predicted to contribute to inter-trimer interactions in the mature MA lattice did not affect viral infectivity or viral replication. These findings suggest that a well-organized mature MA lattice is not essential for viral infection.

The HBR, which includes residues Arg^21^, Lys^25^, Lys^26^, Lys^29^, and Lys^31^, creates a positively charged area on the MA globular domain’s surface that facilitates membrane binding via electrostatic interactions with the acidic headgroups of phospholipids including PI(4,5)P_2_ and PS (*26*, *49*, *50*). The L20K mutation introduces an additional positive charge within the HBR, thereby enhancing its membrane affinity (*34*). Nuclear magnetic resonance (NMR) studies have demonstrated that, when MA binds to PS, there is a favorable interaction between the acidic headgroup of PS and the R42 residue of MA (*14*). The crystal structure of the MA trimer reveals a central pore surrounded by three R42 residues (*15*). The density observed in the trimer center of both immature and mature WT MA lattices indicates the possible presence of a negatively charged entity that could interfere with phospholipid interaction. In the immature L20K/E73K/A82T mutant, the absence of central density, coupled with upward reorientation of R42 in the AlphaFold2 prediction, as well as the E73K mutation, is presumed to enhance interactions with lipid headgroups, consistent with the role of R42 in phospholipid binding. The collective structural modifications induced by L20K and E73K and the repositioning of R42 create a highly positive surface for phospholipid binding and docking, offering an explanation for the increased Gag binding affinity observed for the L20K/E73K/A82T mutant (*32*).

Recent structural analysis suggests that proteolytic cleavage of SP2 from the Gag precursor by PR is required to form a mature MA lattice (*51*). The SP2 peptide was shown to bind to the inter-trimer interface, previously predicted to be a PI(4,5)P_2_ binding pocket (*18*), and contributes to stabilizing the mature MA lattice. This study also reported that fusion kinetics are influenced by the release of SP2. However, previous studies have shown that deletion of SP2 or modifications at the NC-SP2 cleavage site confer only minor defects in virus infectivity (*52*–*54*). The results reported herein also show that formation of a mature MA lattice, presumably required for SP2 binding, is not strictly required for HIV-1 particle infectivity. Thus, while SP2 may contribute to the reorganization of the immature MA lattice to the mature form, further work will be required to fully elucidate the interplay between formation of the mature MA lattice, SP2 binding, and particle infectivity.

## MATERIALS AND METHODS

### Cell lines and plasmids

HeLa cells [obtained from the American Type Culture Collection (ATCC)], human embryonic kidney (HEK) 293T cells (obtained from the ATCC), and TZM-bl cells [obtained from J. C. Kappes, X. Wu, and Tranzyme Inc. through the National Institutes of Health (NIH) AIDS Reagent Program (ARP), Germantown, MD] were maintained in Dulbecco’s modified Eagle’s medium supplemented with 10% fetal bovine serum at 37°C in 5% CO_2_. The H9 T cell line was cultured in RPMI 1640 medium supplemented with 10% FBS at 37°C in 5% CO_2_.

The full-length HIV-1 molecular clone pNL4-3 (*55*) was used in this study. pNL4-3 MA-L20K and L20K/E73K/A82T, and R19L were described previously (*32*, *34*, *48*). We introduced alanine mutations in MA by HiFi DNA Assembly Master Mix (New England Biolabs) according to the manufacturer’s instructions. For biosafety reasons, VLPs for structural analysis were prepared with a pNL4-3 derivative containing inactivating mutations in integrase (IN) and RT (pNL4-3 IN-D116N/RT-D186N) (*56*) or subviral plasmid pCMVNLGagPolRRE, which expresses NL4-3 GagPol from a CMV promoter (*57*). To prepare immature VLPs, the PR active site mutation PR-D25N was introduced into pNL4-3 RT-D186N/IN-D116N or pCMVNLGagPolRRE plasmids. The MA-coding regions of pNL4-3 MA mutants were cloned into pCMVNLGagPolRRE using Bss HII and Spe I restriction sites. The HIV-1 Env expression vectors, pIIINL4env and pIIINL(AD8)env, were described previously (*48*, *58*).

### Antibodies

Anti-Gag (ab63917, anti-Pr55Gag + p24 + p17 Ab) and anti-tubulin Abs (B-5-1-2) were purchased from Abcam and Sigma-Aldrich, respectively. The polyclonal anti-Gag Ab was produced by immunizing rabbits with a recombinant Gag protein. Anti-gp41 (Chessie 8) and polyclonal anti-p17 antiserum (ARP-4811) were obtained through the NIH HIV Reagent Program. The anti-p17 antiserum was produced by immunizing rabbits with a recombinant, histidine-tagged, full-length MA protein (*59*). Monoclonal anti-p24 Ab conjugated with fluorescein isothiocyanate (KC57 clone) was purchased from Beckman Coulter. Additionally, a polyclonal anti-Gag p17 Ab (PAB1178) was obtained from Abnova.

### Preparation of viruses or VLPs

For VLPs used in the structural analysis, HEK293T cells were co-transfected with pCMVGagPolRRE, pIIINL4env, and pSV-Tat (*60*) at a ratio of 16:4:1 using GenJet In Vitro DNA Transfection Reagent (Ver. II) (SignaGen Laboratories). Alternatively, pNL4-3 RT-D186N/IN-D116N and pIIINL(AD8)env were co-transfected at a ratio of 10:1. Culture media from transfected cells were harvested at 48 hours post-transfection and passed through a 0.45-μm polyvinylidene fluoride filter. The VLPs were concentrated by ultracentrifugation through an 8% OptiPrep density gradient (Sigma-Aldrich) (100,000*g*, Sorvall AH-629 rotor) for 1 hour at 4°C. The concentrated VLPs were further purified by ultracentrifugation (120,000*g*, Sorvall TH-660 rotor) through a 10 to 30% OptiPrep gradient for 2.5 hours at 4°C. The opalescent band was harvested, diluted with phosphate-buffered saline (PBS), and ultracentrifuged at 110,000*g* at 4°C for 2 hours. Pelleted particles were resuspended in 5% sucrose/PBS solution and stored at −80°C until use. For cryo-EM grid preparation, a 3-μl aliquot was placed onto a glow-discharged lacy grid (AGS166-3 Lacey Carbon Films on 300 Mesh Copper Grids) and plunge-frozen in liquid ethane using the Leica EM GP 2 system for back-side blotting.

### Membrane stripping assay

The HEK293T or HeLa cell lines were transfected with WT or mutant pNL4-3 molecular clones using Lipofectamine 2000 (Invitrogen). Viruses were incubated with various concentrations of NP-40 for 5 min. Following incubation, the viruses were purified by ultracentrifugation through 20% sucrose cushions (60,000*g*) for 45 min at 4°C. For sedimentation analysis, the concentrated viruses incubated with 0.075% NP-40 were subjected to ultracentrifugation through a 10 to 70% sucrose gradient (60,000*g*) for 16 hours. Alternatively, the viral membrane was stripped by a “spin-through” detergent-treatment method with minor modification (*37*). Briefly, the concentrated viruses were subjected to ultracentrifugation (60,000*g* for 2 hours at 4°C) through a layer of PBS or 1% Triton X-100 into a linear OptiPrep density gradient (10 to 60%). The core fraction was identified on the basis of density and by RT assay as described previously (*61*). Aliquots from each fraction were lysed and subjected to Western blot analysis.

### Western blotting

Cell- and virus-associated proteins were solubilized in lysis buffer [30 mM NaCl, 50 mM tris-HCl (pH 7.5), 0.5% Triton X-100, 10 mM iodoacetamide, and cOmplete protease inhibitor (Roche)]. Lysates boiled in 6× loading buffer [7 ml of 0.5 M tris-HCl/0.4% SDS, 3.8 g of glycerol, 1 g of SDS, 0.93 g of dithiothreitol (DTT), and 1.2 mg of bromophenol blue] were subjected to SDS-PAGE and transferred to polyvinylidene disulfide membranes (Merck Millipore). After blocking the membranes with Azure Fluorescent Blot Blocking Buffer (Azure Biosystems) or 5% milk/TBS-T, the membranes were probed with the indicated Abs (1:5000 to 10,000) at 4°C overnight and then incubated for 1 hour with species-specific AzureSpectra Fluorescent Secondary Antibodies (Azure Biosystems) or horseradish peroxidase (HRP)–conjugated secondary Abs (GE Healthcare). After the final washes, bands were detected by fluorescence with a Sapphire Biomolecular imager (Azure Biosystems). Chemiluminescence signals were detected by SuperSignal West Pico PLUS Chemiluminescent Substrate (Thermo Fisher Scientific). Quantification was performed using ImageJ software.

For Western blotting of WT MA or MA mutant proteins (fig. S13, A and B), these proteins were first expressed in *Escherichia coli* Rosetta 2 (DE3) cells by inducing with 0.1 mM isopropyl-β-d-thiogalactopyranoside (IPTG) at 37°C for 3 hours in Luria-Bertani medium. Following expression, cells were harvested by centrifugation and resuspended in lysis buffer [50 mM tris-HCl (pH 8.0), 150 mM NaCl, and 0.1% Triton X-100]. The lysate was clarified by centrifugation at 20,000*g* for 30 min using an SW-32 rotor. Both the soluble supernatant and insoluble pellet fractions were analyzed via SDS-PAGE to assess the solubility of the expressed protein. Proteins were then transferred to a nitrocellulose membrane, which was probed with a polyclonal anti-Gag p17 Ab (PAB1178). After primary Ab incubation, the membrane was washed thoroughly and incubated with an anti-mouse HRP-conjugated secondary Ab (Sigma-Aldrich, A0168) at a 1:5000 dilution for 1 hour at room temperature. Protein bands were visualized using Clarity Western ECL substrate (Bio-Rad) according to the manufacturer’s protocol.

### Single-round infectivity assay

Single-round infectivity assays were performed as previously described (*61*). The amount of virus in the supernatant was quantified by an RT assay, performed as described previously (*61*). TZM-bl cells (1.0 × 10^4^ cells) in 96-well plates were incubated with RT-normalized virus stocks. At 48 hours postinfection, luciferase activity was measured using the Britelite plus reporter gene assay system (PerkinElmer) and GloMax Navigator microplate luminometer (Promega).

### Virus replication kinetics analysis

Virus replication was monitored in H9 T cell line as previously described with minor modifications (*62*). H9 cells were incubated with the indicated pNL4-3 MA variants (1.0 μg DNA/1.0 × 10^6^ cells) in the presence of DEAE-dextran (700 μg/ml) at 37°C for 15 min. Transfected cells were cultured in 24-well plates. Aliquots of supernatants were collected to measure RT activity, and cells were split 1:1 every other day with fresh medium.

### Protein purification and cryo-EM

WT MA and MA L20K/E73K/A82T mutant genes were cloned into the pSY5 vector (*63*), which includes an N-terminal 8-histidine tag followed by a human rhinovirus (HRV) 3C PR cleavage site (LeuGluValLeuPheGln↓GlyPro). The plasmid was transformed into *E. coli* strain BL21 (DE3). The proteins were expressed overnight (18°C) in terrific broth medium supplemented with 0.4% (v/v) glycerol, following induction by 0.2 mM IPTG at a cell density characterized by an optical density at 600 nm of 0.8 to 0.9. The resultant cell pellets (10 g) were resuspended in binding buffer of 50 ml [20 mM Hepes, 500 mM NaCl, 20 mM imidazole, and 1 mM TCEP (pH 7.7)] supplemented with Triton X-100 [0.01% (v/v)], PR inhibitor cocktail (Set III, EDTA-free, Calbiochem) and benzonase (2 μl of 10,000 U/μl, Merck). Cell lysis was performed using an ultrasonic cell disrupter Vibra-Cell (Sonics). The proteins bound to a Ni–nitrilotriacetic acid (NTA) affinity chromatography column (HisTrap FF GE Healthcare), washed with binding buffer containing 20 mM tris-HCl (pH 8.0), 1 M NaCl, 1 M (NH_4_)_2_SO_4_, 30 mM imidazole, and 5 mM β-mercaptoethanol. The target protein was eluted with 15 ml of elution buffer composed of 20 mM tris-HCl (pH 8.0), 500 mM NaCl, 1 M (NH_4_)_2_SO_4_, 5 mM β-mercaptoethanol, and 500 mM imidazole. The eluted fraction was concentrated to ~2 ml, and the His tag was cleaved with His-tagged HRV 3C PR (0.1 mg/ml) during an overnight dialysis against a buffer containing 20 mM tris-HCl (pH 8.0), 500 mM NaCl, 5% (v/v) glycerol, and 1 mM DTT. Following cleavage, the reaction mixture was diluted to a total volume of 50 ml. The untagged MA protein, along with the His-tagged HRV 3C PR and uncleaved MA, was separated using reverse Ni-NTA affinity chromatography. The untagged MA protein was collected as the flow-through fraction. To denature the protein, 2 ml of the flow-through was combined with 10 ml of denaturation buffer [20 mM tris-HCl (pH 8.0), 1 M NaCl, 1 M (NH_4_)_2_SO_4_, 1 mM DTT, and 6 M urea] and allowed to incubate overnight during dialysis. Following this, the sample was concentrated to a final volume of 1 ml, and size exclusion chromatography (SEC) was performed using a Superdex 200 10/300 increase column (GE Healthcare, Little Chalfont, UK). The column was equilibrated with SEC buffer [20 mM tris-HCl (pH 8.0), 150 mM NaCl, and 1 mM DTT] and run on an Akta FPLC system (GE Healthcare) at a flow rate of 0.2 ml/min. Peak fractions containing the refolded MA protein were pooled and concentrated (2000 MWCO Amicon Ultra Centrifugal Filter concentrator) for cryo-EM analysis (fig. S13, D and E).

Three microliters of purified WT or mutant MA protein (1 mg/ml) was applied to glow-discharged Lacey carbon-coated copper grids (300 mesh, Agar Scientific). The grids were blotted under 100% humidity and plunge-frozen into liquid ethane using a Vitrobot Mark IV (Thermo Fisher Scientific). Following vitrification, the grids were clipped and screened using a Glacios cryo–electron microscope (Thermo Fisher Scientific).

### Cryo-ET and subtomogram averaging

The cryo-ET data were acquired with 300-kV Titan Krios (eBIC Krios III), using Thermo Fisher Scientific Falcon 4i detector & Selectris X energy filter with 5-eV window. Tomographic tilt series were collected with tilt angles ranging from −60° to +60°, in 3° increments, following a dose-symmetric scheme with a group size of 3, using Tomo 5 (Thermo Fisher Scientific). The effective magnification was set to ×81,000, resulting in 1.50 Å/pixel on the specimen. The nominal defocus for each tilt series ranged from 1.5 to 5.5 μm. Movies were captured for each tilt, consisting of 10 frames in counting mode. Dose requirement is 3 e/Å^2^ per tilt, resulting in total cumulative 123 e/Å^2^ per series. Details of all data acquisition settings can be found in table S1.

Workflow for data processing is presented in fig. S3. Frames were motion-corrected using MotionCor2 (*64*). Fiducial alignment of tilt stacks was done using IMOD (*65*). Subtomogram alignment and averaging were conducted using emClarity (*39*, *66*) and Relion (*38*).

For CA of immature mutant WT and mutant particles, we selected particles from 6× binned, non-contrast transfer function (CTF)–corrected tomograms template matching through emClarity/1.5.0.2, using a 28-Å low-pass filter on the EMD-8403 (*67*). The magpiEM tool (https://github.com/fnight128/MagpiEM) was used for cleaning based on the geometric restraints of the CA lattice. Three-dimensional (3D) alignment and averaging for hexamer CA was incrementally refined from 6× to 1× binning in emClarity/1.5.3.10, preserving C6 symmetry.

For MA of immature mutant WT and mutant particles, the immature lattice structure from PDB 7OVQ (*18*) served as the initial template. We generated an extended 400-Å-wide lattice map for MA template matching on 6× binned, CTF-corrected tomograms using emClarity/1.5.3.10, with a 28-Å low-pass filter applied. The magpiEM tool was also used for data cleaning on the basis of geometric constraints, particularly between the bilipid and CA layers. Top-view subtomograms, identified by a tilt angle under 45° relative to the beam, were selected for iterative alignment, beginning from 6× binning down to 4× binning. Subsequently, these subtomograms were subjected to global in-plane and local out-of-plane refinements at their 4× binned top-view alignment. This refined positioning also included further cleaning to remove duplicates and ensure adherence to local MA lattice constraints. The further cleaned MA subtomograms were then prepared for averaging in Relion 4, enforcing C3 symmetry after global refinement at bin4 and local refinement at bin2.

For mature WT MA, we used a mature, 400-Å-wide lattice model extended from PDB 7OVR (*18*) as a template for matching on 6× binned, non-CTF-corrected tomograms using emClarity/1.5.0.2 with a 28-Å low-pass filter to the template. The magpiEM script was used to select MA subtomogram matches on the basis of geometric constraints beneath the bilipid layer. Criteria for further selecting trimeric MA structures ensured the exclusion of unreliable peaks. This selection step prepared the dataset for further refinement in Relion 4.0.0, where 3D refinement was performed at gradually decreasing bin levels, maintaining C3 symmetry throughout.

### MA lattice arrangement analysis

In the comparative analysis for subtomograms that were mapped back, we define the distance and tilt angle for trimer-trimer pairings within the MA lattice. The lattice maps of CA hexamers and MA trimers were generated using the ArtiaX for UCSF Chimera X (*68*, *69*). To facilitate a reasonable comparison between the immature mutant and WT MA lattice maps, the density map threshold for MA is calculated using a 1% false discovery rate (*70*).

### Radial density profile analysis

We enhanced the signal from the central slice of a VLP by summing it with 10 neighboring slices. The positions of the OL within the slice were identified to serve as a reference point for subsequent analysis. To accurately locate the OL, we manually marked points along its perimeter, fitted these points to a circle path, and generated normal line density profiles perpendicular to this path. The position of the OL in each profile was determined by identifying local minima around the plotted circle. These profiles were then averaged, aligning them on the basis of the OL position to ensure consistency. We accumulated a substantial number of VLP average profiles (over 20 VLPs for each sample) to ensure the robustness of our analysis. The final average profile was displayed with error bars to illustrate the membrane-associated density representation of the sample. Additionally, a box plot of the distance between different valleys and the OL was created to further analyze the data (fig. S9).

### HIV-1 lipid membrane modeling

In preparation for the atomistic MD simulations of an MA lattice embedded in an HIV-1 lipid membrane with native composition, we modeled the model membrane by the following procedure: First, we derived the stoichiometric ratios of lipid species corresponding to native HIV-1 lipidomics (*71*); for the purpose of modeling an asymmetric membrane, the intravirion and outervirion leaflet ratios for each lipid species were estimated from united atom-based simulations of an HIV-1 lipid vesicle (*72*). The resulting stoichiometric ratios between different lipid species are shown in table S2.

We previously showed that the viral membrane of a spherical virion is asymmetric due to the differences in curvature between the OL and IL of the virion (*72*). Therefore, to build a flat asymmetric lipid bilayer of native HIV-1 lipid composition, we followed the procedure proposed by Pastor and co-workers (*73*). For the latter, we prepared two symmetric lipid bilayers using CHARMM-GUI (*74*, *75*): (i) a lipid bilayer corresponding to the extravirion leaflet composition (table S2) and (ii) a lipid bilayer corresponding to the intravirion leaflet composition (table S2). Each symmetric membrane was solvated and ionized by adding NaCl ions to neutralize the charge of the system and further solvated in a water box containing a salt concentration of 150 mM NaCl. The resulting symmetric membranes were then equilibrated following the CHARMM-GUI Membrane Builder protocol (*76*, *77*). Namely, the system was minimized using a conjugate gradient scheme until the gradient converged below 10 kcal/mol per square angstrom, followed by a lipid tail–melting procedure where the lipids molecules were allowed to reorganize and repack the membrane for 2 ns at constant temperature of 310 K and constant pressure of 1 bar and contain 905,725 and 831,950 atoms for the intravirion and extravirion symmetric leaflet systems, respectively; water molecules are kept out of the membrane hydrophobic core by applying external forces on the oxygen atoms of water molecules that penetrate in the membrane. Subsequently, the system is equilibrated for 50 ns at 310 K and 1 bar and contains approximately 830,000 particles; constant area pressure control was used with a total cross-sectional area of 100,997 and 86,201 Å^2^ for the intravirion and extravirion symmetric leaflet systems, respectively; symmetric membrane simulations were performed in NAMD 2.15b14 (table S3, simulations 1 and 2). All membrane MD simulations used the hydrogen mass repartition scheme (*78*), which enables a 4-fs timestep for time propagation. During equilibration simulations, temperature was maintained at 310 K using a Langevin thermostat with a thermal coupling constant of 1 ps^−1^ and a pressure of 1 atm via a Nose-Hoover barostat with a period of 100 fs and decay time of 50 fs.

Following equilibration of the symmetric lipid bilayers, the coordinates of the lipid molecules, ionic molecules, and water molecules associated with the IL of the intravirion symmetric membrane and the OL of the extravirion symmetric membrane were extracted and merged into a single asymmetric membrane [following the work of Pastor and co-workers (*73*)]. The latter procedure conserves the ion and water molecules absorbed to lipid headgroups in each leaflet, as well as water solvation shells, in the asymmetric membrane. Due to the differences in lipid per area and lipid compositions in the intravirion and extravirion leaflets, the intravirion leaflet resulted in a larger area than the extravirion leaflet and was thus trimmed to fit the dimensions of extravirion leaflet while maintaining the molar fraction ratios for each lipid species. The resulting asymmetric membrane extended over an area of 29.4 nm by 29.4 nm with a resulting composition detailed on table S2.

After building the HIV-1 flat asymmetric lipid membrane, we performed lipid tail melting and equilibration following the aforementioned procedure implemented for the symmetric membranes (*76*). Subsequently, we performed three independent replicates of 1-μs canonical MD simulation in the constant number of particles, pressure, and temperate (NPT) ensemble at a temperature of 310 K and pressure of 1 atm using the Langevin thermostat and Nose-Hoover barostat with the same parameters as above, keeping the ratio of *X*-*Y* unit cell constant while allowing fluctuation in all axes. Long-range electrostatics were calculated using the particle mesh Ewald method (*79*) with a short-range cutoff of 12 Å and switching parameter of 10 Å (table S3, simulation 3). All simulations were performed using NAMD 2.15alpha2 and NAMD 3beta6 (*80*).

### MA lattice modeling

A model of the full-length MA was derived from the x-ray crystal structure of the MA trimer (PDB ID 7TBP) (*40*), complemented with the terminal MA residues 109 to 115 from the NMR myrMA monomer structure (PDB ID 2LYB) (*14*). Thirteen MA trimers were rigid-body fitted into EM densities for the WT immature MA, L20K/E73K/A82T immature MA, and WT mature MA densities to build the respective MA lattice models; each model consisting of a 12-nucleotide oligomer of trimers (Fig. 5I). The three systems were then prepared for MD simulations by adding hydrogens to MA according to the protonation state of the amino acids at pH 7.0 as predicted by propKa3 (*81*, *82*). The protein was then solvated with TIP3P water molecules into a periodic box and ionized with Na^+^ and Cl^−^ ions to achieve a concentration of 150 mM and neutral charge using the solvate, cionize, and autoionize plugins in the Visual Molecular Dynamics (VMD) software (*83*).

The rigid body fitted MA lattices were then refined via Molecular Dynamics Flexible Fitting (MDFF) (*84*) by running MD in an NPT ensemble and using the cryo-EM density as a grid-based biasing potential coupled to the protein heavy atoms, effectively biasing their movement to fit the electron density. MDFF was performed for 10 ns with a coupling gird scaling factor of 0.3 kcal/mol/amu L20K MA mature and immature lattices, as well as the initial model for the L20K/E73K/A82T MA mature lattice simulations, were built from the mature or immature WT MA lattices by introducing the mutations with the Mutator plugin in VMD (*85*). The structure of each MDFF-fitted MA lattice was further refined using an automated structure refinement procedure (*86*) using RosettaScripts (*87*) as preparation for deposition to the protein data bank. Final refined structures are deposited with accession codes PDB ID 9EK3 and PDB ID 9EK2 for the immature WT and L20K/E73K/A82T MA assembly, respectively, and PDB ID 9EK1 for the mature WT MA assembly.

### MA embedded in an authentic HIV-1 lipid membrane complex

After deriving models for MA lattices guided by the electron density, we used the protein coordinates and merged them with the equilibrated coordinates of the previously prepared HIV-1 lipid membrane. First, we placed the MA lattice 10 nm from the intravirion leaflet and outside of the simulation box; subsequently, we solvated the MA lattice and ionized with Na^+^ and Cl^−^ ions to a 150 mM concentration. Then, to embed MA into the lipid membrane, we applied a constant velocity pulling to the last carbon of the MA myristoyl tail toward the hydrophobic core of the lipid bilayer at a rate of 5 Å/ns by applying successive harmonic restraints with a spring constant of 0.5 kcal/mol per square angstrom in 100 successive 0.1-ns MD simulations until all myristoyl tails were embedded in the lipid bilayer hydrophobic core. These pull-and-wait simulations (*88*, *89*) were performed in an NPT ensemble with constant area pressure control and a pressure of 1 atm via a Nose-Hoover barostat with a period of 200 fs and decay time of 100 fs, while the temperature was maintained at 310 K using a Langevin thermostat using a high Langevin coupling constant of 100 ps^−1^ to dampen the dynamics of the lipid membrane during myristoyl tail insertion. This effectively restricted the axial movement of the lipid membrane without adding constraints to the positions of the lipid headgroups.

After the myristoyl tail insertion procedure described above, the Langevin coupling constant was reduced to 10 ps^−1^ over 20 ns and, subsequently, to 5 ps^−1^ and 1 ps^−1^ over the same period. This approach reduced drag and allowed the MA lattice to be adsorbed into the lipid membrane without restricting protein and lipid dynamics. This procedure was applied to all MA lattices to build mature and immature MA-membrane complexes for WT MA, L20K, and L20K/E73K/A82T mutant MA, resulting in six MA-membrane complex models indicated in table S3 (simulations 4 to 9) as well as the four salt-bridge–perturbing MA mutations: R19A, R19L, E41A, and E51A (simulations 10 to 13).

### Protein-lipid interaction analysis

Each immature MA-membrane complex was subjected to three replicates of canonical MD simulations in an NPT ensemble for 1 μs (table S3, simulations 4 to 6). Throughout each trajectory, we tracked the instantaneous lateral displacement of lipids in the intravirion leaflet using a vector velocity field (*41*). This method discretizes the space into a grid of 2D voxels and tracks the movement of lipids though the membrane by calculating the displacement of the center of mass of the group of lipids ${\vec{X}}_{i}$ in the *i*th voxel from a time *t* to a time *t* + *dt* as a vector

$${\vec{u}}_{i\left(t\right)}={\vec{X}}_{i\left(t+\mathrm{dt}\right)}-{\vec{X}}_{i\left(t\right)}$$

which is then visualized using streamlines. We used the lipid headgroup atoms to calculate centers of mass on each voxel a grid spacing of 10 Å to which ensured an average of ~5 lipid molecules per voxel and a *dt* = 0.2 ns as indicated in the work by Chavent *et al.* (*41*) (fig. S6 and movie S1). As indicated in aforementioned work, to obtain a smooth transition of velocity vectors through time, we preprocessed the 1-μs trajectories using a low-pass filter of the coordinates using the gmx_filter tool in GROMACS version 2024.3 (*90*). Time averages of lipid displacement are then calculated on a per-voxel basis to identify static regions in the membrane (Fig. 4B and fig. S6D).

Lipid-protein contacts were analyzed throughout the simulation in VMD (*83*) using in-house Tool Command Language (*91*) scripts. These contacts were measured over each frame of the simulation trajectory using a distance threshold of 3.5 Å between protein and lipid headgroup heavy atoms. Contact occupancies were measured for each MA residue and every lipid species in the intravirion leaflet of the membrane and averaged over the 39 monomers in the 12-nucleotide oligomer of trimers assembly. This approach ensures that, if a lipid-protein interaction has high occupancy in one monomer but is not present in the others, then its average contribution to the MA-membrane interactions is reduced. The highest MA-lipid occupancy for each MA residue was then represented visually in the protein structure model of an MA trimer (Fig. 4, F to H), permitting the identification of high occupancy regions in both the WT MA lattice and L20K/E73K/A82T MA lattice.

Lipid headgroup occupancies were calculated for all lipid species and per-lipid species through the 1-μs trajectories (simulations 4 to 6) with a spatial resolution of 1 Å by using the volmap pluigin in VMD (*83*), and the voltool plugin was used to generate a difference map between the lipid headgroup occupancies in the L20K/E72K/A82T MA and WT MA simulations. Occupancy maps are visualized at the 0.25 isovalue representing sites where lipid headgroups localize for over 25% of the simulation.

### Perturbative MD simulations of MA lattices adsorption by lipid bilayers

The stability of mature MA lattices was probed in a novel perturbative approach based on controlled damping of Langevin dynamics during protein-membrane adsorption, as described below. The main idea of our method consists of perturbing the molecular environment surrounding MA, by transferring MA from a liquid solvent (water and ions) to a complex lipid environment. To control the temperature in MD simulations, the movement of particles in the system is coupled to a thermal bath at a constant temperature T by using a thermostat. In the case of the widely used Langevin thermostat (*92*), the dynamics of the *i*th particle in the ensemble, with mass *m*_*i*_ and position $\vec{r_{i}}$, is described by

$$m_{i}\frac{\partial^{2}\vec{r_{i}}}{\partial t^{2}}=-{\vec{\nabla }}_{i}V-\gamma m_{i}\frac{\partial \vec{r_{i}}}{\partial t}+\vec{R_{i}}$$

where *V* is the molecular potential, $\gamma m_{i}\frac{\partial \vec{r_{i}}}{\partial t}$ is a dissipative drag force with friction coefficient γ, and $\vec{R_{i}}$ is a random force due to thermal motion of the particles in the coupled thermal bath, modeled as a gaussian noise with zero mean and variance $\sigma^{2}=2m_{i}\gamma k_{B}T/\Delta t$. The dissipative drag force dampens the momentum of particles, and the friction coefficient γ effectively controls the viscosity of the solvent. If γ is too large, then the system is overdamped and obeys Brownian dynamics. In contrast, if γ is too small, then the system is underdamped and may have trouble dissipating heat in a short simulation time, with the limit case γ = 0 representing a system with no temperature coupling.

The Langevin thermostat guarantees ergodicity and, in equilibrium simulations, evolution toward canonical ensemble distributions after enough sampling regardless of the friction coefficient (*93*). However, previous computational studies have shown that in nonequilibrium processes, variations in the Langevin friction coefficient can cause differences in macroscopic observables [material hardness (*94*) and diffusion coefficients (*95*)] and microscopic structure [crystal lattice plastic deformation (*94*)].

Protein-membrane adsorption is a dynamic process, in which the both protein and lipids can undergo structural changes (*96*, *97*): Localization of protein can induce the formation of lipid raft domains in the membrane (*98*, *99*), while protein conformational changes can be promoted by adsorption into the membrane (*100*–*102*). For HIV-1 MA, protein adsorption is driven by electrostatic interactions between negatively charged lipid headgroups and positively charged residues in the surface of MA and is enhanced by the hydrophobic interactions caused by myristoil-tail insertion in the membrane (*103*, *104*).

In our approach, we conduct nonequilibrium MD simulations of MA adsorption to the lipid membrane by gradually decreasing the Langevin friction coefficient in three successive stages from 10 to 5 to 1 ps^−1^, each lasting 20 ns. This method maintains a constant temperature and ensures proper heat dissipation throughout the adsorption process, while the sequential reduction in the friction coefficient allows for the gradual displacement and interaction of the MA lattice with the lipid membrane via electrostatic interactions. In this process, as the mature MA lattice approaches the membrane, it is gradually perturbed by the changes in chemical environment and decreasing drag forces until it reaches the adsorbed-equilibrium conformation. Structural stability of the MA lattice through the simulation depends on the strength of interactions between MA trimers and their ability to withstand the variations in chemical environment and drag forces.

Each mature MA lattice was probed via six replicas of perturbation/adsorption experiments (table S3, simulations 7 to 13), in which the L20K, L20K/E73K/A82T, R19A, R19L, E41A, or E51A mutations were introduced to the mature WT MA system after embedding the myristoyl tail in the membrane and reionizing the system to charge neutrality. The membrane and MA lattice were then progressively brought together by the previously described adsorption/perturbation simulations, with constant ratio pressure control and a pressure of 1 atm via a Nose-Hoover barostat with a period of 20 ps and decay time of 10 ps. The temperature was maintained at 310 K using a Langevin thermostat with Langevin friction coefficient reduced from 10 to 5 and 1 ps^−1^ over periods of 20 ns for a full simulation time of 60 ns. This approach progressively changes the chemical environment that the MA lattice is exposed to, allowing us to study the structural stability of the lattice and energetic favorability of MA-MA interfaces.

Through the simulations, we calculate the distance between neighboring MA trimers as

$$d_{\mathrm{ij}}=\left\|{\vec{COM}}_{i}-{\vec{COM}}_{j}\right\|$$

where ${\vec{\mathrm{COM}}}_{i}$ is the center of mass of the *i*th MA trimer in the lattice. We identified lattice-breaking events as those where the trimer-trimer distance was greater than the maximum trimer-trimer distance measured from the cryo-EM density and WT MA equilibration (*d*_*ij*_ > 55.0 Å). Similarity between the trimer-trimer distance distributions was measured using the Jensen-Shannon distance metric (*43*, *44*). This is a symmetric similarity metric bound between 0 and 1, where two probability distributions will have a distance further from 0 the more dissimilar they are.

We estimated the binding affinity between pairs of neighboring MA trimers by using the molecular mechanics generalized solvent surface area (MM-GBSA) (*45*, *46*, *105*) method. MM-GBSA allows the calculation of free binding energies post-simulation by calculating interaction energy between two groups of atoms from the molecular mechanics potentials and approximating bulk effects in the long-range electrostatic and van der Waals interactions with the generalized born and surface area continuum solvation model. MM-PBSA/GBSA has been used recently to estimate protein-protein binding affinities in molecular complexes (*47*, *106*, *107*). We calculate the difference between MA-MA trimer binding affinities of mutant MA compared to WT MA from the MA lattice perturbation simulations using the MMPBSA.py tool (*45*) available in Amber22 (*108*), using the igb = 5 generalized Born solvation model with 150 mM salt concentration.

## Supplementary Materials

### The PDF file includes:

Figs. S1 to S13 Tables S1 to S4 References
