# Supplementary material for: Structural maturation of the matrix lattice is not required for HIV-1 particle infectivity
Source: Sci Adv. 2025 May 9;11(19):eadv4356. doi: 10.1126/sciadv.adv4356 (PMC12063641; doi:10.1126/sciadv.adv4356)
Supplement: Supplementary file 1 — Figs. S1 to S13 Tables S1 to S4 References [file sciadv.adv4356_sm.pdf]

Science Advances
AAAS

Supplementary Materials for

Structural maturation of the matrix lattice is not required for HIV-1 particle infectivity

Long Chen *et al.*

Corresponding author: Peijun Zhang, peijun.zhang@strubi.ox.ac.uk; Eric O. Freed, efreed@nih.gov; Juan R. Perilla, jperilla@udel.edu

Sci. Adv. **11**, eadv4356 (2025)
DOI: 10.1126/sciadv.adv4356

The PDF file includes:

Figs. S1 to S13
Tables S1 to S4
References

Other Supplementary Material for this manuscript includes the following:

Movies S1 to S5

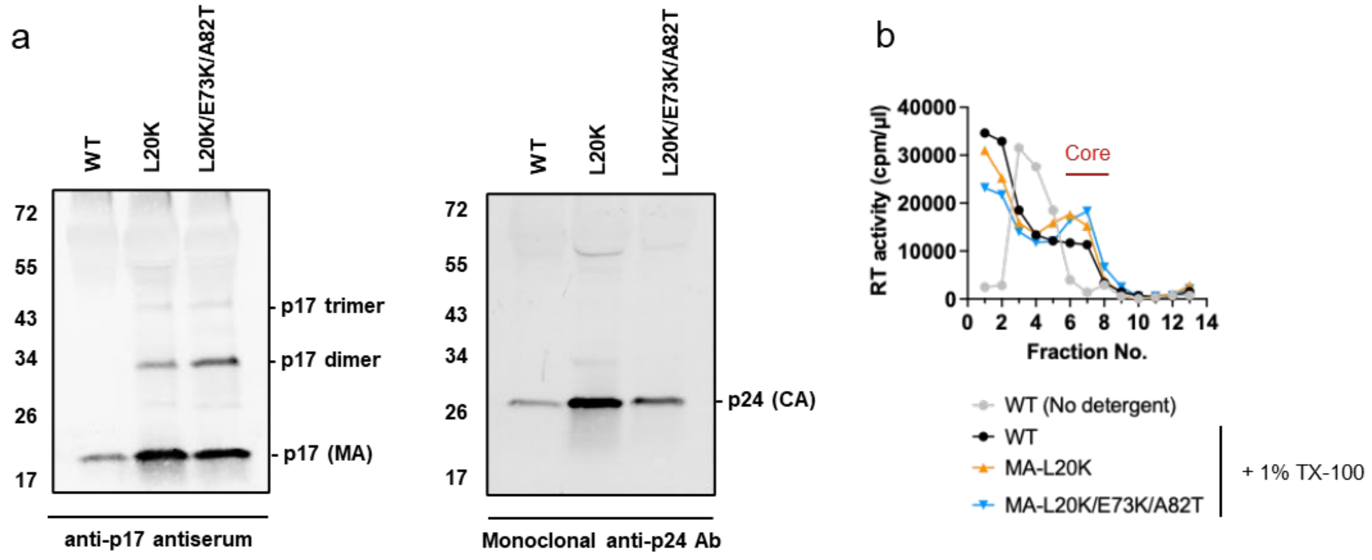

Figure S1 | Western blotting of viral lysates with monocloncal anti-p24 Ab. a) virion-associated proteins derived from 293T cells transfected with WT or MA-mutant pNL4-3 molecular clones were probed with anti-p17 antiserum or monoclonal anti-p24 Ab. b) Representative results of RT assay from three independent experiments related to Fig. 1g are shown.

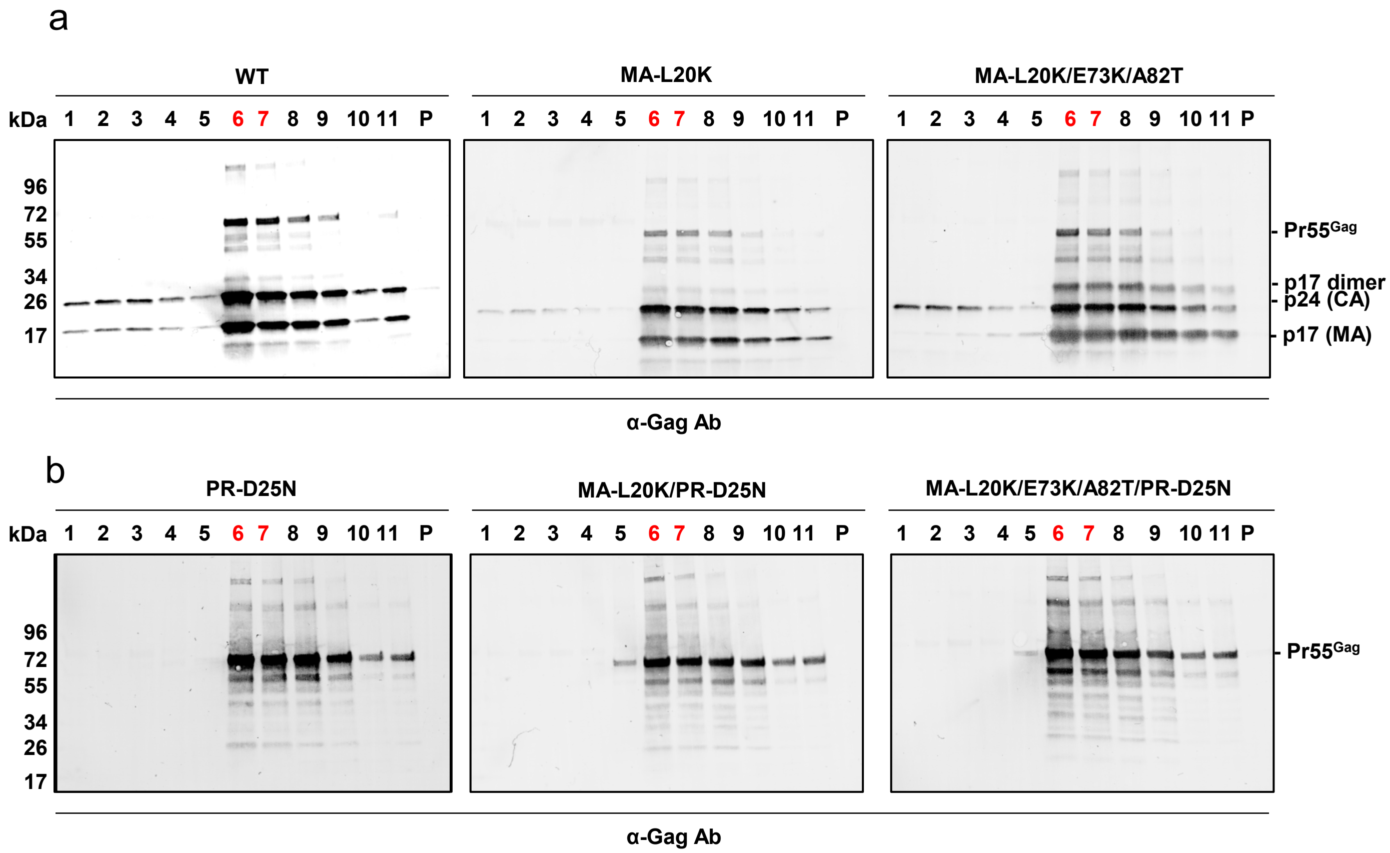

Figure S2 | Sedimentation analysis of mature and immature VLPs. a-b) Sedimentation analysis of (a) NL4-3 RT-D186N/IN-D116N pseudotyped with NL(AD8) Env (mature virions) and (b) NL4-3 PR-D25N/RT-D186N/IN-D116N pseudotyped with NL(AD8) Env (immature virions) in 10-30% optiprep gradient without any detergent. The western blots of the gradient fractions using anti-Gag Ab for both the WT and mutant MA. P, pellet fraction. Fractions 6 and 7 were pooled and subjected to structural analysis (related to Fig. S10).

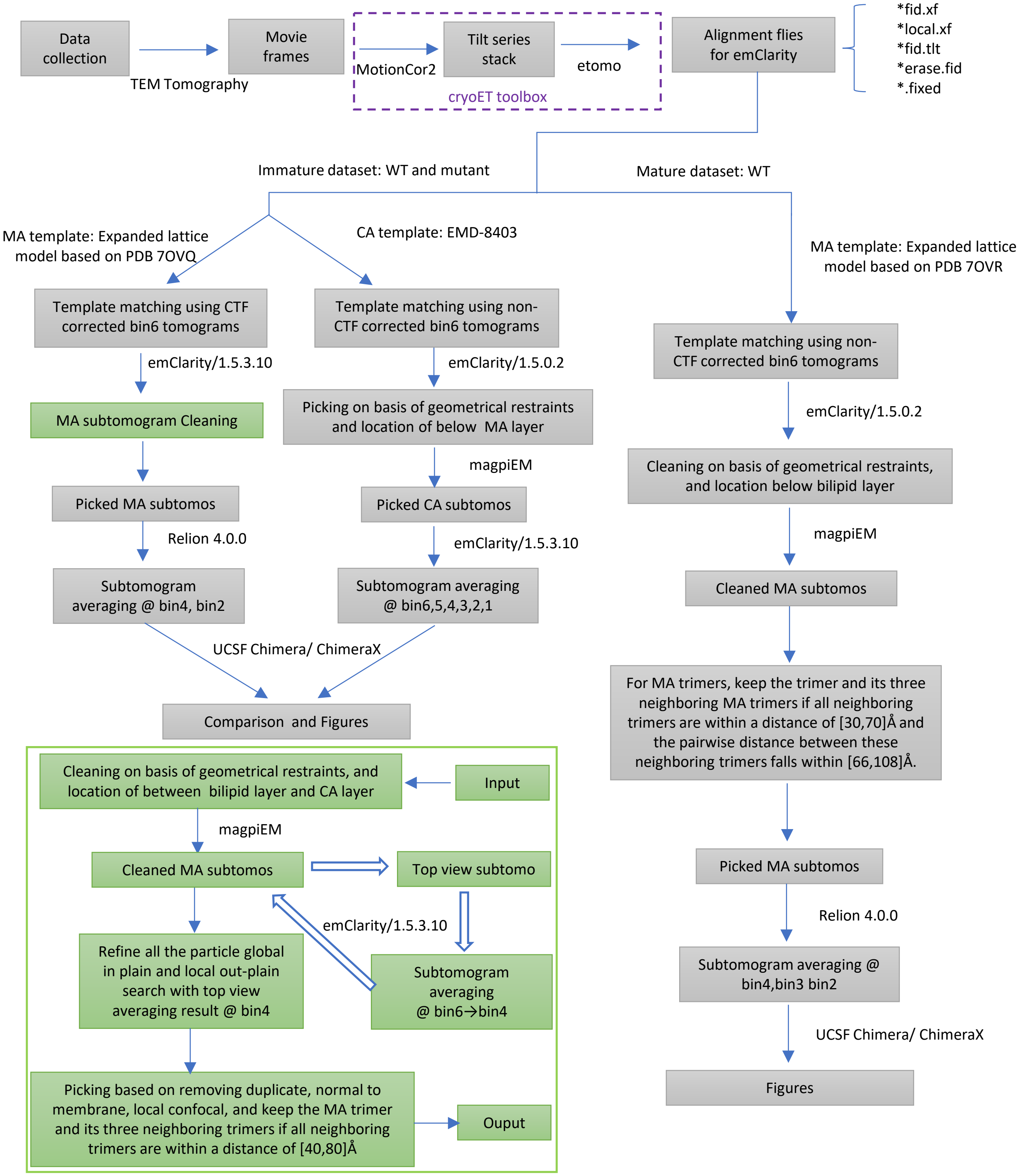
Data collection
TEM Tomography
Movie frames
MotionCor2
Tilt series stack
etomo
cryoET toolbox
Alignment flies for emClarity
*fid.xf
*local.xf
*fid.tlt
*erase.fid
*.fixed
Immature dataset: WT and mutant
Mature dataset: WT
MA template: Expanded lattice model based on PDB 7OVQ
CA template: EMD-8403
MA template: Expanded lattice model based on PDB 7OVR
Template matching using CTF corrected bin6 tomograms
Template matching using non-CTF corrected bin6 tomograms
Template matching using non-CTF corrected bin6 tomograms
emClarity/1.5.3.10
emClarity/1.5.0.2
emClarity/1.5.0.2
MA subtomogram Cleaning
Picking on basis of geometrical restraints and location of below MA layer
Cleaning on basis of geometrical restraints, and location below bilipid layer
magpiEM
Picked MA subtomos
Picked CA subtomos
magpiEM
Relion 4.0.0
emClarity/1.5.3.10
Cleaned MA subtomos
Subtomogram averaging @ bin4, bin2
Subtomogram averaging @ bin6,5,4,3,2,1
For MA trimers, keep the trimer and its three neighboring MA trimers if all neighboring trimers are within a distance of [30,70]Å and the pairwise distance between these neighboring trimers falls within [66,108]Å.
UCSF Chimera/ ChimeraX
Comparison and Figures
Cleaning on basis of geometrical restraints, and location of between bilipid layer and CA layer
Input
magpiEM
Cleaned MA subtomos
Top view subtomo
emClarity/1.5.3.10
Refine all the particle global in plain and local out-plain search with top view averaging result @ bin4
Subtomogram averaging @ bin6→bin4
Picking based on removing duplicate, normal to membrane, local confocal, and keep the MA trimer and its three neighboring trimers if all neighboring trimers are within a distance of [40,80]Å
Ouput
Picked MA subtomos
Relion 4.0.0
Subtomogram averaging @ bin4,bin3 bin2
UCSF Chimera/ ChimeraX
Figures

Figure S3 | Workflow for data processing. TEM Tomography 5 was utilized to capture Falcon4 tilt-series. On-the-fly pre-processing was conducted via the CryoET Toolbox (https://github.com/ffyr2w/cet_toolbox). This process included frame motion correction via MotionCor2 [63] and fiducial marker-based alignment through etomo [64]. The resulting alignment files were then transferred to emClarity for template matching [39, 65]. Template matching outcomes was cleaned for both CA and MA based on geometric constraints with in-house-developed script magpiEM (https://github.com/fnight128/MagpiEM). The cleaned subtomogram selection dataset was randomly split into two subsets for parallel processing. The gold-standard Fourier shell correlation was computed from separate even and odd halves.

For immature CA, we selected particles from 6x binned, non-CTF-corrected tomograms through emClarity/1.5.0.2 template matching, employing a 28 Å low-pass filter on the EMD-8403 template [66]. Hexamer CA's iterative alignment was conducted from 6x to 1x binning using emClarity/1.5.3.10, maintaining C6 symmetry throughout.

The immature MA analysis began with the PDB 7OVQ [18] template to create a 400Å-wide lattice map for template matching on 6x binned, CTF-corrected tomograms using emClarity/1.5.3.10, also with a 28 Å low-pass filter. The cleaning was undertaken by geometric constraints, focusing on the inter layer between the bilipid and CA layers with magpiEM. Due to false positive peaks induced by the missing wedge from side views, top view subtomograms were selected firstly, restricting the choice to those peaks with a tilt angle of less than 45 degrees relative to the beam direction. Subsequent averaging for top view subtomograms was performed from bin6 to bin4. We refined all subtomograms globally in-plane and locally out-of-plane, ensuring top view coordinates aligned with those refined at bin4. MA subtomograms were picked by removing duplicates and satisfying local lattice constrain, readying them for subtomogram averaging in Relion 4.0.0 with global refinement at bin4 and local refinement at bin2 with C3 being enforced [38].

For mature wild-type MA, a mature expanded 400Å-wide lattice model based on PDB 7OVR was used as the template [18]. Template matching is operated on 6x binned, non-CTF-corrected tomograms in emClarity/1.5.0.2, using a 28 Å low-pass filter. The matching result was cleaned based on geometric restraints and location below the bilipid layer with magpiEM. Further cleaning of trimeric MA was guided by criteria that retained a trimer and its three neighboring trimers if all neighboring trimers fell within a distance of [30,70] Å and the pairwise distances between these neighboring trimers ranged from [66,108] Å. This process removed unreliable particle peak, yielding a dataset of cleaned subtomograms. These subtomograms were further processed using Relion 4.0.0, where subtomogram averaging was initially performed at bin4 with global refinement, then followed by local refinement at bin3 and bin2, enforcing C3 symmetry throughout.

ChimeraX [68] and ArtiaX [67] were used for visualization and analysis of the density maps, supplemented by false discovery rate analyses [69].

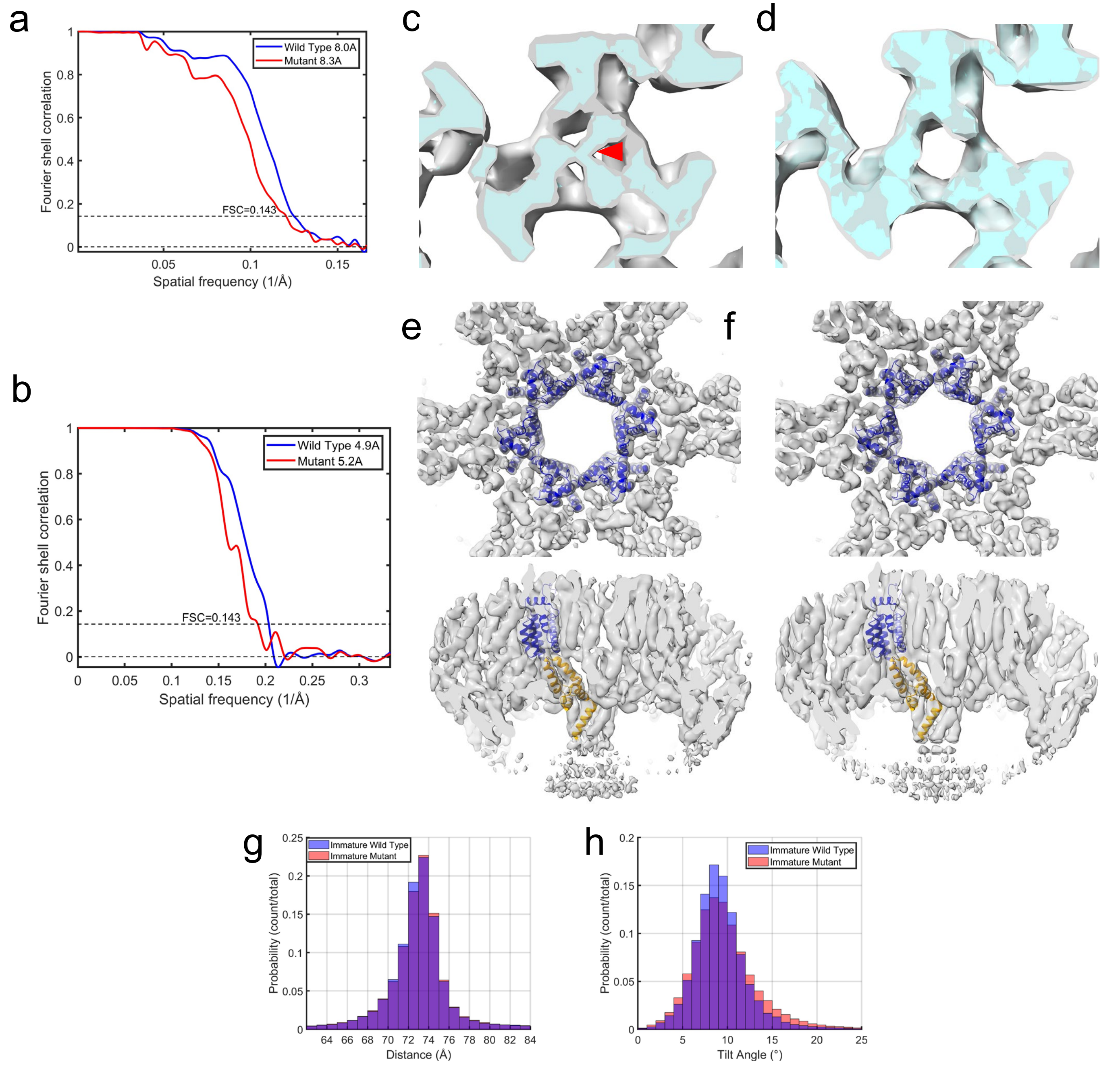

Figure S4 | Structures of immature MA and CA. a-b) Fourier shell correlation (FSC) plots for the MA trimer (a) and CA hexamer (b) from both immature wild type and mutant, with resolutions indicated at the FSC value of 0.143. c-d) Final refined maps for MA wild type (c) and mutant (d) with C1 symmetry, displaying distinct central differences. The top view of the confidence map, calculated at a 1% false discovery rate [69], is shown in cyan, superimposed on the gray MA trimer map. (e-f) The difference map from Fig. 3c is overlaid on the wild-type MA trimer map. e-f) CA hexamer maps for the wild type (e) and mutant (f), displayed in top and side views, are superimposed with the model (PDB 5L93) [111]. The N-terminal domain (NTD) and C-terminal domain (CTD) of CA are colored blue and orange, respectively. (g-h) Comparisons of distances and tilt angles between hexamer-hexamer pairings in tomographic reconstructions reveal that the CA-CA distance for the WT averages 72.9 Å with a standard deviation of 2.9 Å, compared to the mutant's mean of 73.0 Å and standard deviation of 2.9 Å. Tilt angles for the WT and mutant are 9.1° (SD 2.7°) and 9.4° (SD 3.3°) respectively.

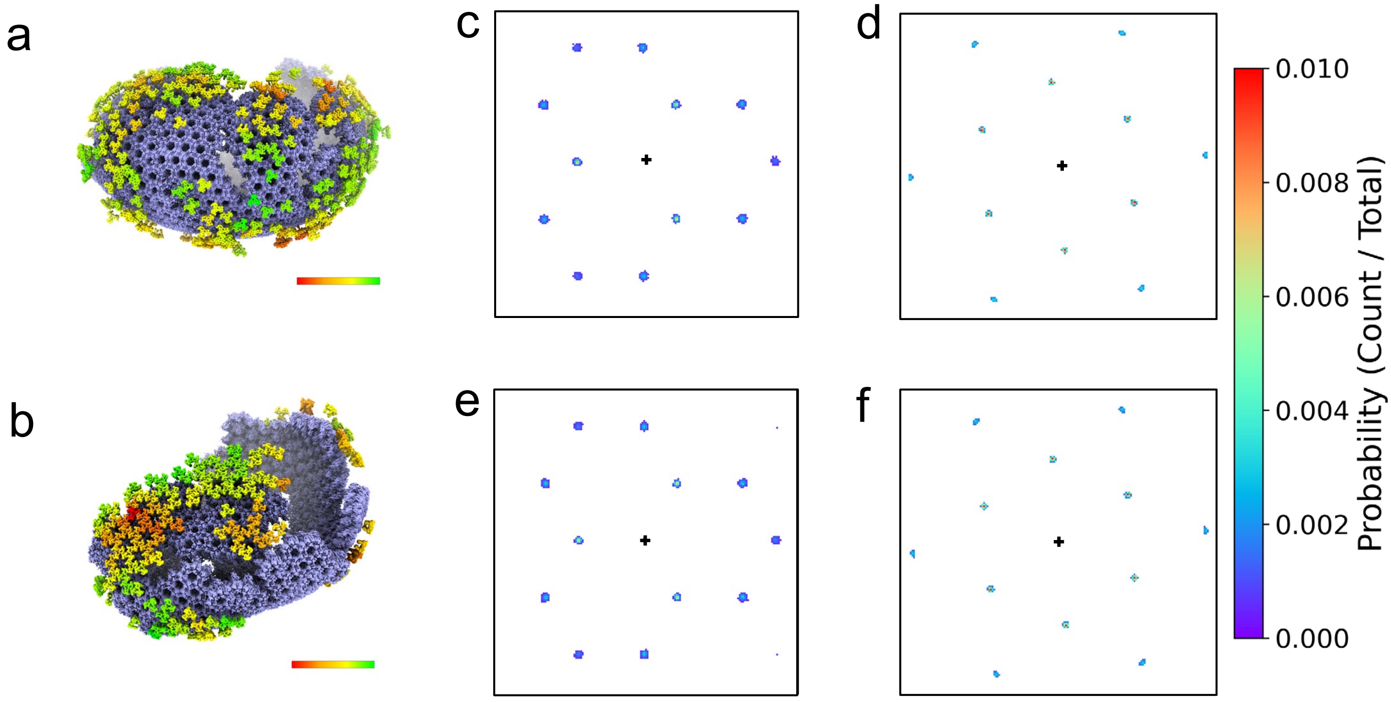

Figure S5 | lattice regularity comparison of MA and CA in immature wild type and L20K/E73K/A82T mutant VLPs. a-b) Lattice maps of MA trimer (colored) and CA hexamer (blue) from an example tomogram of WT (a) and mutant (b) VLPs. MA trimers are colored by cross correlation values with the average MA trimer map, from red (lower) to green (higher), with ranges of 1.501e6 to 1.512e6 for wild type and 1.459e6 to 1.471e6 for mutant. c-f) Localization plots for MA trimers and CA hexamers illustrate the positions of neighboring units around a central trimer or hexamer (black cross). In WT VLPs, there are 18,262 MA trimers (c) and 30,782 CA hexamers (d). In mutant VLPs, there are 8,600 MA trimers (e) and 30,646 CA hexamers (f). The color gradient in the heatmaps, from blue to red, indicates the increasing probability for the occurrence of neighbouring trimers or hexamers as per the color key.

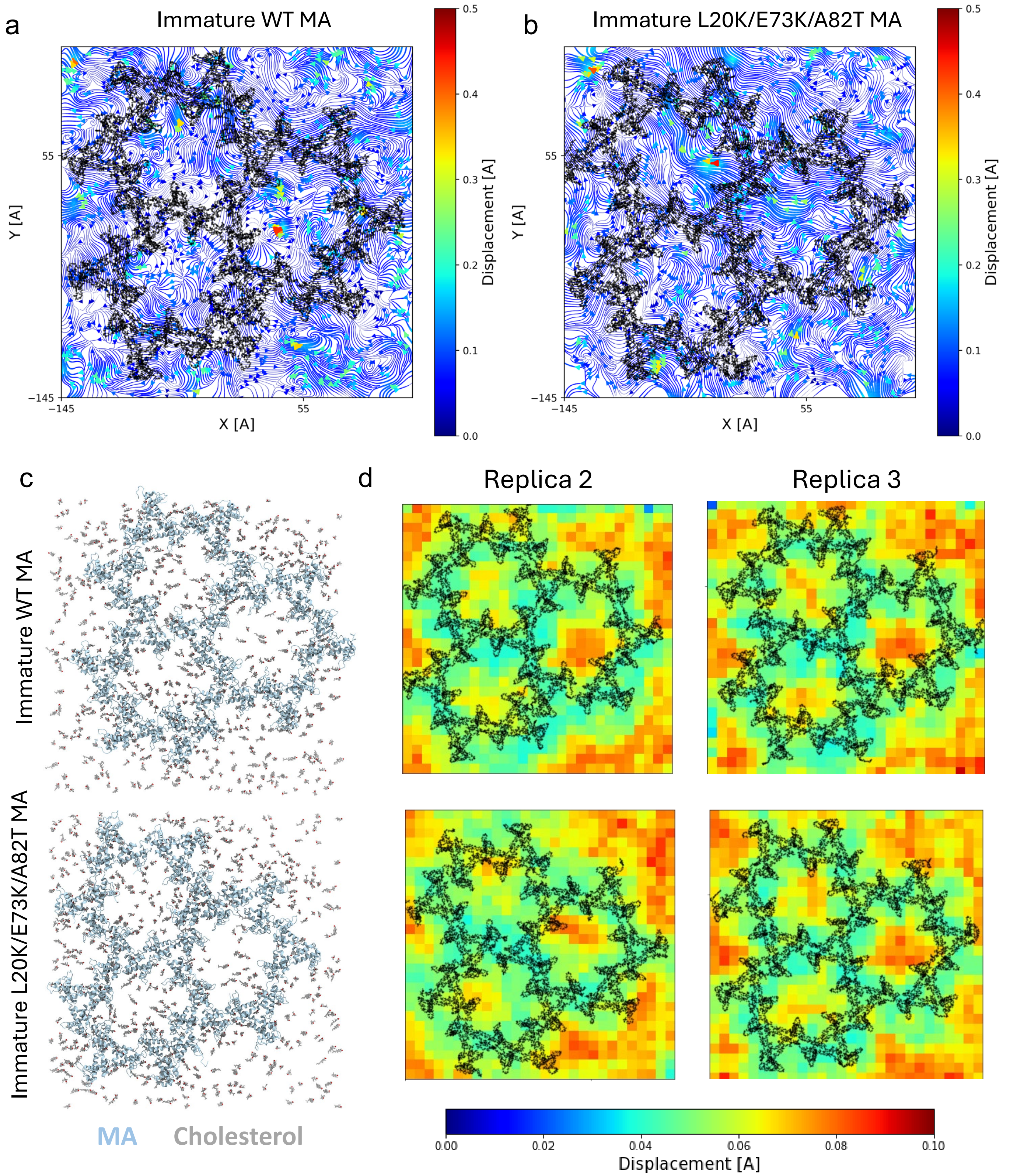

Figure S6 | Instantaneous lateral lipid displacement visualization. a-b) Streamline visualization of the instantaneous lateral displacement of lipid headgroups in the intravirion leaflet in the presence of the immature WT MA lattice (a) or the immature L20K/E73K/A82T MA lattice. The intravirion leaflet is discretized in a 2D grid with 10 Å spacing, and the displacement of the center of mass of lipid headgroups in each voxel is tracked at 0.2 ns intervals. The streamlines are the tangential curves to displacement vectors in the grid and represent the continuous "flow" patterns of the lipids. Streamlines are colored by the magnitude of the displacement at each grid point. The MA lattice backbone is colored in black. Instantaneous displacement through the 1 µs trajectory is presented in Movie S1.

(c) The immature WT MA lattice (top) and L20K/E73K/A82TA MA lattice (bottom), along with the position of cholesterol molecules in the intravirion leaflet constrain lipid dynamics, resulting in static regions in the membrane.

(d) Time average map of lipid displacement over 1 µs for the second and third MD replica simulations of the viral lipid membrane in complex with the immature WT MA lattice (top) or the immature L20K/E73K/A82T mutant MA lattice (bottom). The average MA position over the trajectory is colored in black. Replica 1 is presented in Figure 4b.

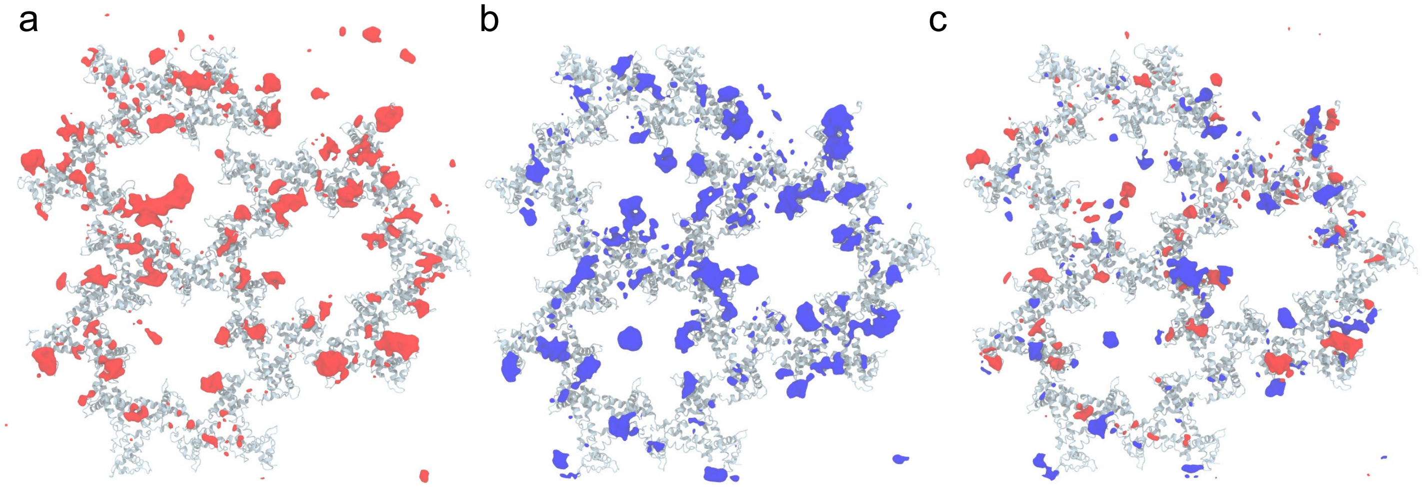

Figure S7 | Lipid headgroup occupancies in presence of immature MA. a-b) Occupancy maps for lipid headgroups through 1 μs of MD simulation for the WT MA (a) or L20K/E73K/A82T MA (b) at isovalue >25%. c) Lipid occupancy difference map, calculated as L20K/E73K/A82T MA – WT MA. Red volumes represent regions present in the WT MA lipid occupancy map and not the L20K/E73K/A82T MA lipid occupancy map. Blue volumes represent regions present in the L20K/E73K/A82T MA lipid occupancy map and not the WT MA lipid occupancy map. Immature MA lattice is shown in light blue in all panels.

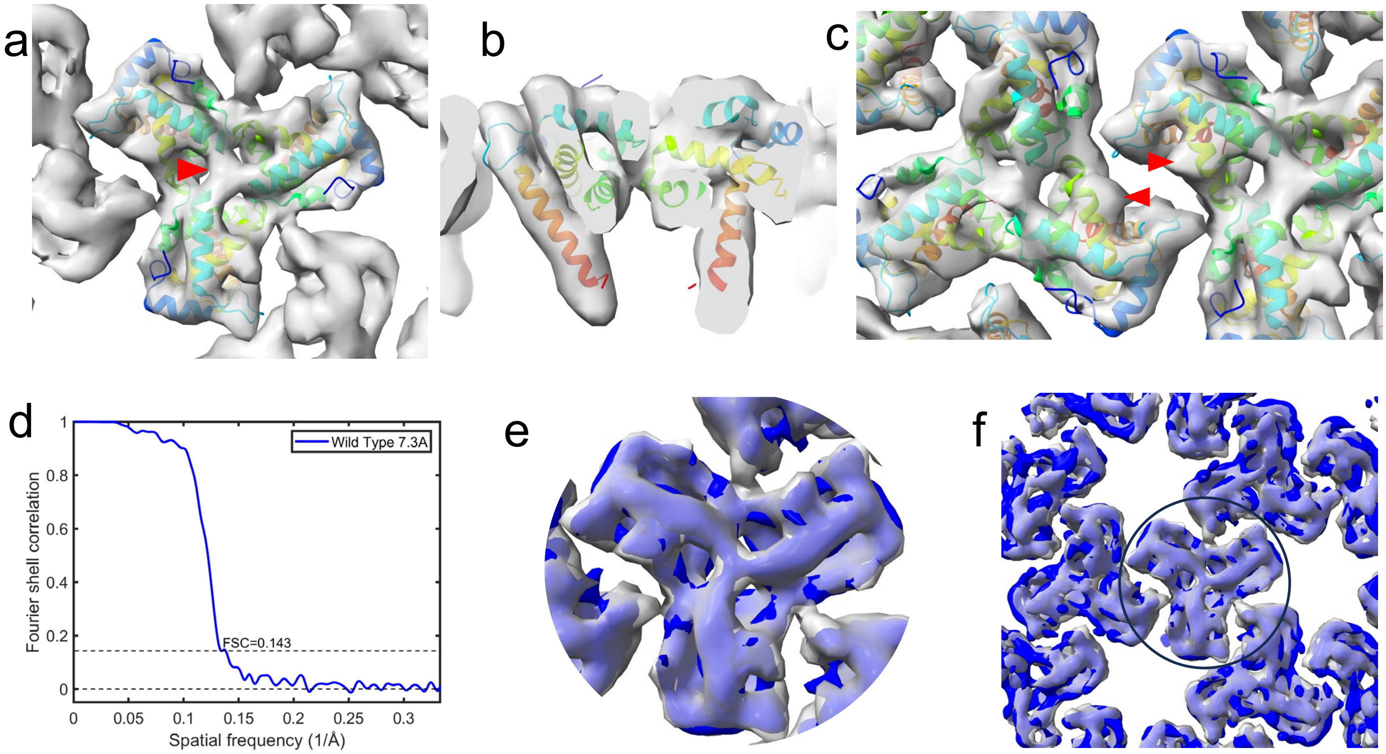

Figure S8 | Subtomogram averaging and FSC of mature wild type MA lattice. a-b) The subtomogram averaging map of the wild type MA trimer is represented in gray isosurface, viewed from the top towards the center of the virus and from the side perpendicular to the membrane. The molecular model of 7OVR [18] is superimposed as a rigid body, color-coded from blue at the N-terminus to red at the C-terminus. c) Density at the PI(4,5)P2 binding site is highlighted by red arrowheads. d) FSC curve assessing the resolution of the MA lattice structure, indicating resolution values at the 0.143 threshold. e-f) The mature MA trimer (e) and its lattice (f) map are overlaid with the the previously published EMD-13088 map [18]. Our MA density is shown in grey and the density of EMD-13088 is in blue.

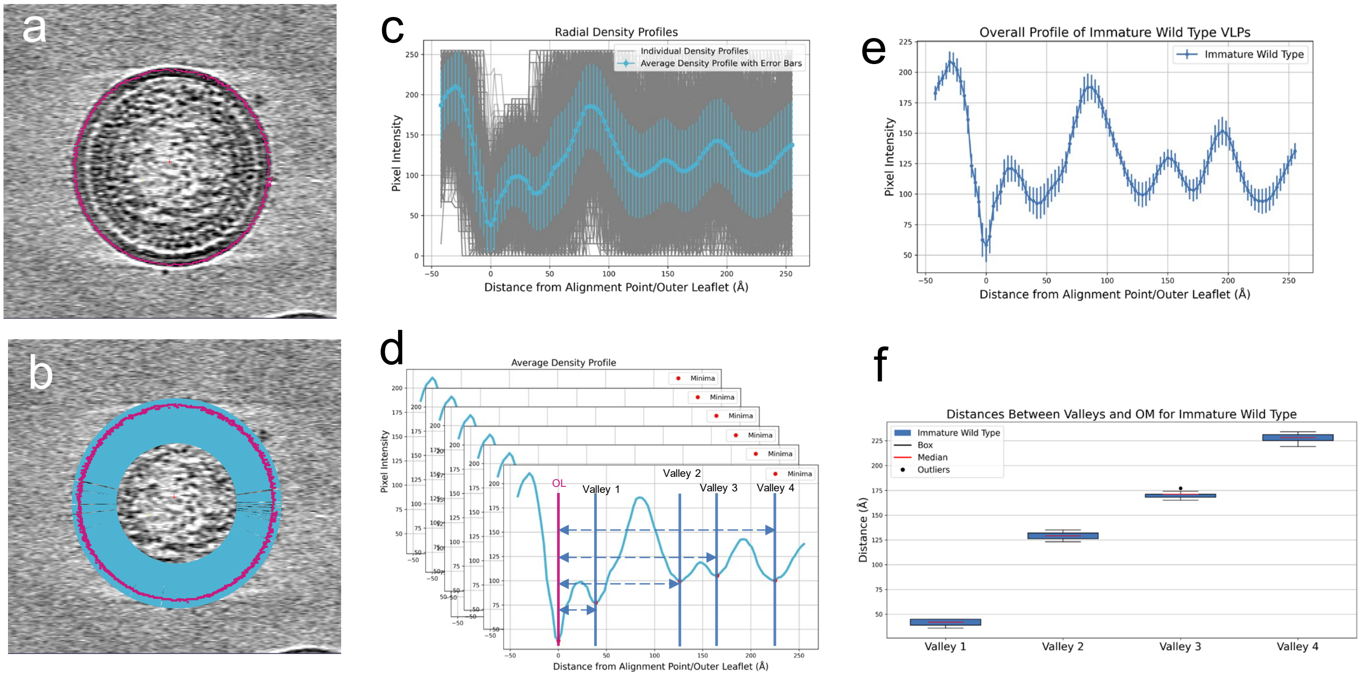

Figure S9 | Profile analysis method: Outer membrane-aligned profiling and averaging. a) Identify the position of the outer leaflet (OL). b) Generate the density profiles perpendicular to OL. c) Compute the average across all profiles aligned based on the OL position. d) Accumulate a sufficient number of VLP average profiles for robust analysis. e) Calculate the overall average profile, presented with error bars. f) Plot a box plot to analyze the distances between various valleys and the OL.

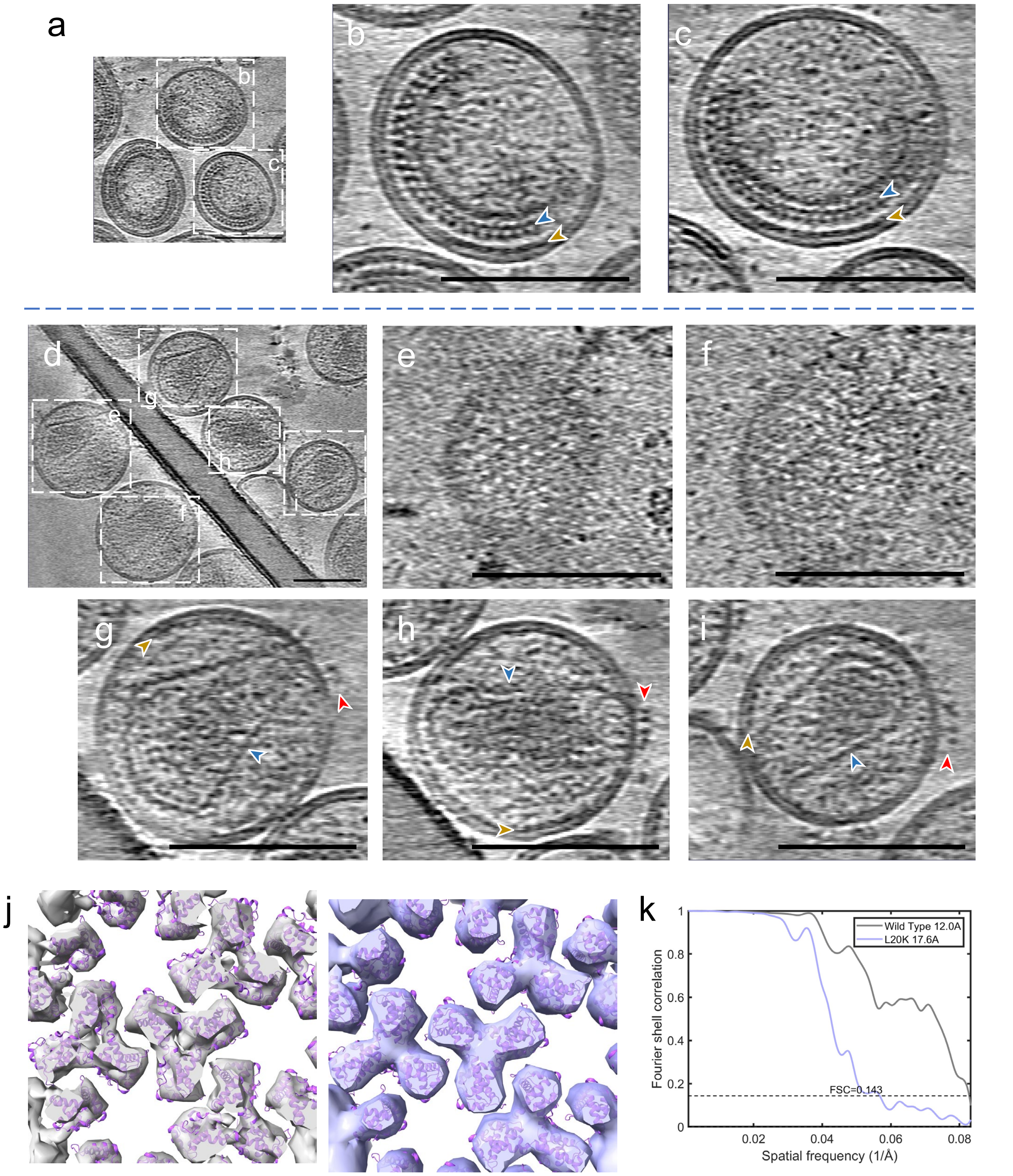

Figure S10 | CryoET of immature and mature MA L20K viruses. a) Representative central slice of immature L20K particles, enhanced by summing 10 adjacent slices. b-c) Detailed views of the central sections from the boxed regions b and c in panel a, similarly enhanced, exhibit the lattice pattern associated with the inner membrane, brown arrowheads mark the positions of the MA lattice, blue arrowheads indicate the CA lattice. d) Representative central slice of mature L20K particles, enhanced by summing 10 neighboring slices. e-f) Magnified top view of slices near the inner membrane surface from boxed areas e and f in panel d, showing lattice patterns. g-i) Magnified central slices from boxed areas g to i in panel d, also enhanced by summing 10 neighboring slices, showing the lattice pattern beneath the inner membrane. Brown arrowheads indicate the MA lattice, blue arrowheads point to the capsid, and red arrowheads highlight the Env glycoproteins. Scale bar: 100nm. j) Mature WT MA lattice (left) and the L20K MA lattice (right) map at bin4 are superimposed with the 7OVR model. k) FSC plots of the MA WT and L20K lattice structures (both bin4), with resolution values indicated at the 0.143 threshold.

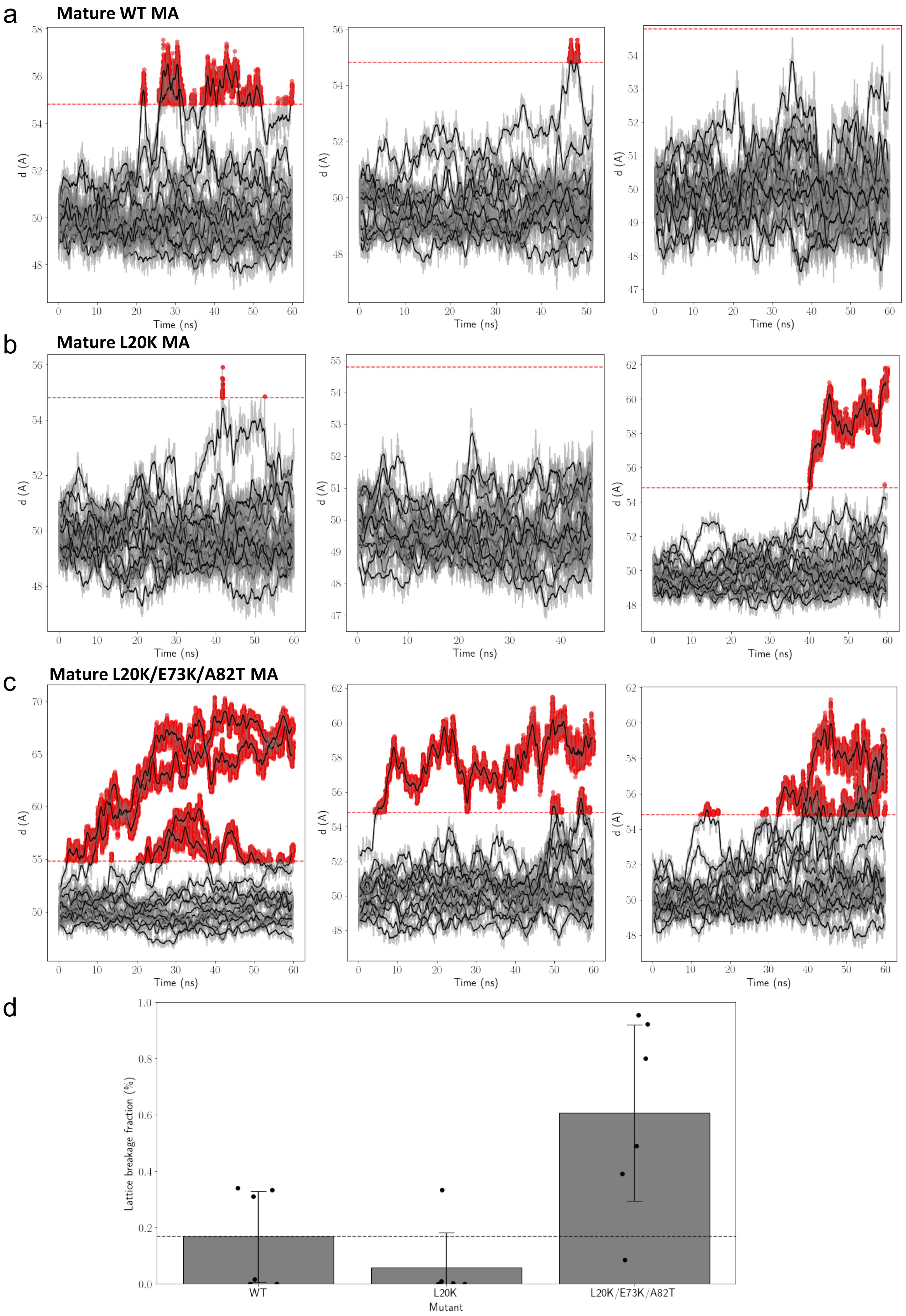

Figure S11 | MA trimer-trimer distance traces from perturbation simulations. a) mature WT MA lattice b) mature L20K MA lattice and c) mature L20K/E73K/A82T MA lattice. Trimers separating in lattice breaking event are colored in red. 3 out of 6 replicas performed are shown. d) Fraction of lattice breakage events though the 6 replicas.

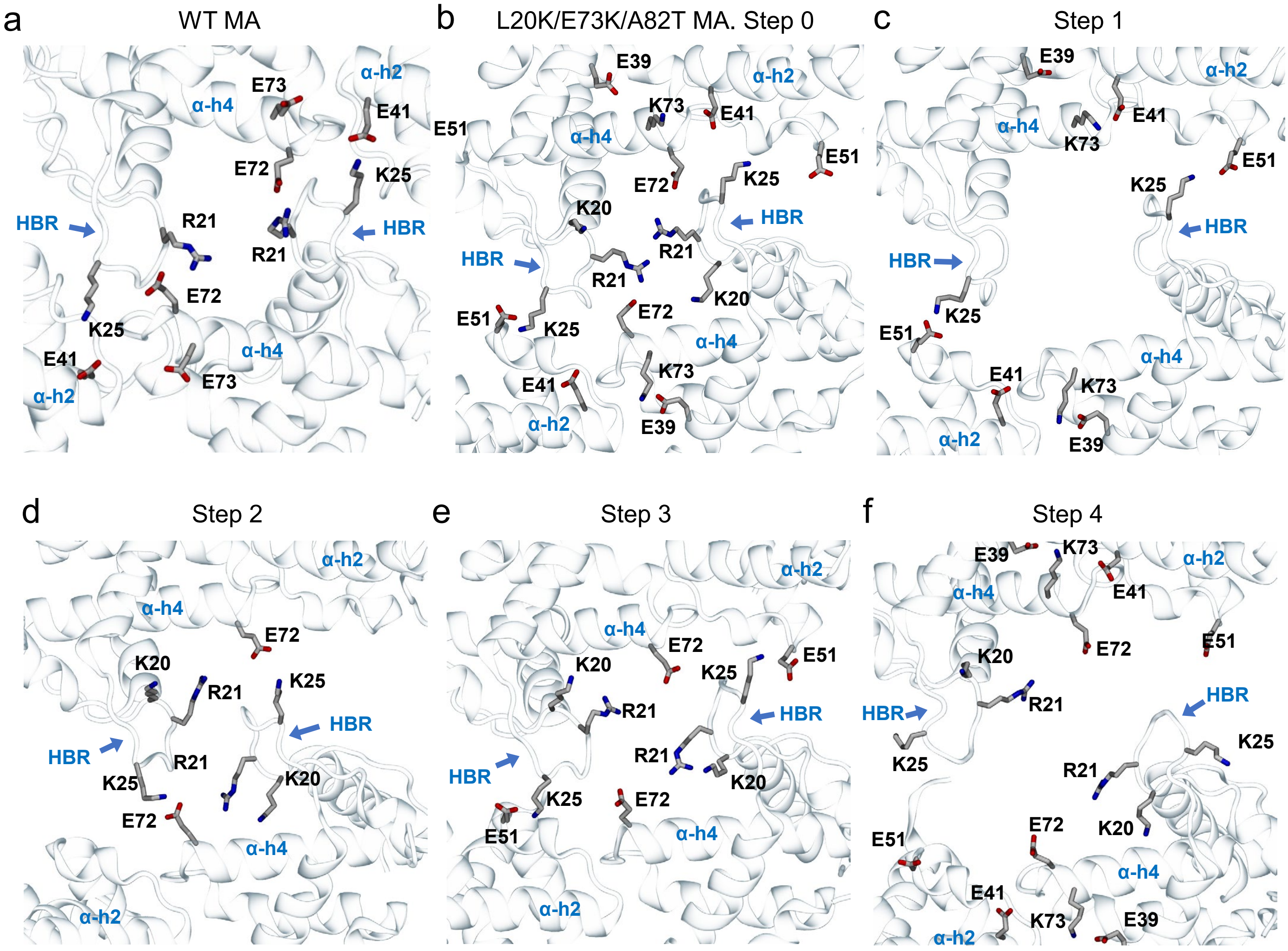

Figure S12 | Allosteric disruption of the mature MA inter-trimer interface by the L20K/E73K mutations. a) Reference salt-bridge network at the MA inter-trimer interface for mature WT MA. b-f) Stepwise process for disruption of the MA inter-trimer salt-bridge network. b) E73K interacts with E39 and E41, impeding the salt bridge formation between K25 and E41. c) K25 instead attempts to form salt bridge interactions with E51 and E72. d) E72 is now attracted by positively charged L20K and interacts with R21 in the same monomer. e) R25 forms the only inter-trimer interaction with E51 while other salt bridges are formed intra-trimer. f) Small displacements of the monomers break R51-E51 and the dimer interface is disrupted.

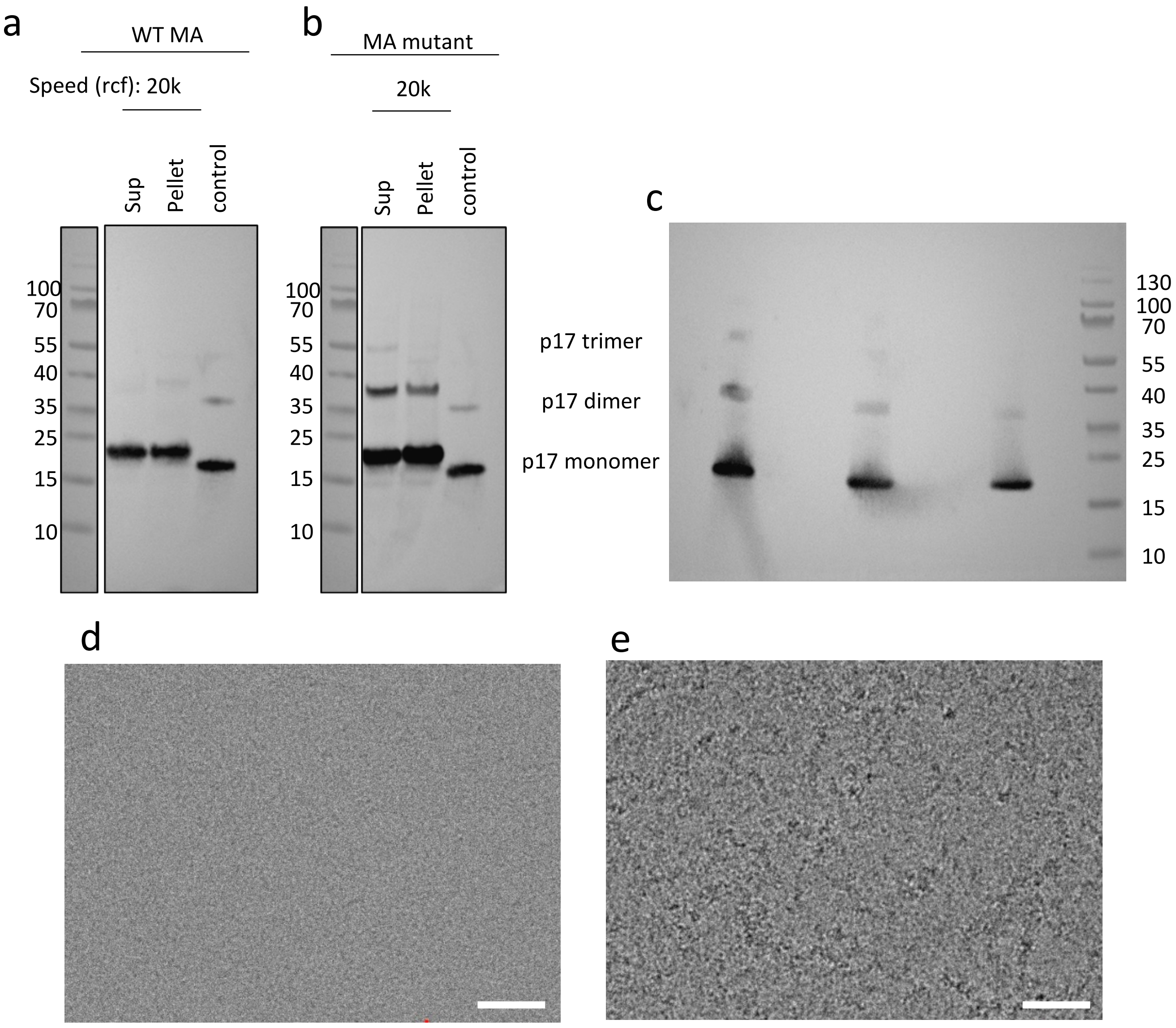

Figure S13 | Purification of WT and L20K/E73K/A82T MA. a-b) Western blot analysis of cell lysates expressing N-terminal 8-histidine tagged WT and L20K/E73K/A82T mutant MA using a polyclonal anti-Gag p17 antibody (PAB1178) at a 1:2500 dilution. Sup: Supernatant, Control: Recombinant MA mutant protein. A total of 10 µg of protein was loaded per well. c) SDS-PAGE gel of purified L20K/E73K/A82T MA, with 1, 0.5 and 0.25 [illegible]g protein loaded. d-e) Transmission electron micrographs of WT MA (d) and L20K/E73K/A82T MA mutant proteins (e). Scale bar 50 nm.

Table S1 | Cryo-EM data acquisition and image processing.

*85 out of 171 total virus particles were randomly selected STA analysis of the CA.

Sample	Immature WT		Immature L20K/E73K/A82T		Mature WT	Mature L20K/E73K/A82T
**Data acquisition**						
Microscope	FEI Titan Krios		FEI Titan Krios		FEI Titan Krios	FEI Titan Krios
Energy-filter slit width (eV)	10		10		10	10
Detector	Falcon4		Falcon4		Falcon4	Falcon4
Pixel size (A)	1.50		1.50		1.50	1.50
Defocus range (microns)	-1.5 to -5.5		-1.5 to -5.5		-1.5 to -5.5	-1.5 to -5.5
Acquisition scheme	-60°/60°, 3° dose symmetric		-60°/60°, 3° dose symmetric		-60°/60°, 3° Dose symmetric	-60°/60°, 3° dose symmetric
Total Dose (electrons/A2)	123		123		123	123
Frame number	10		10		10	10
Tomogram number	89		78		88	93
**Image processing**						
Virus particles	MA	CA	MA	CA	MA	MA
	171	85/171*	109	109	343	428
Subtomogram	18262	30782	8600	30646	47770	Null
Symmetry	C3	C6	C3	C6	C3	Null
Resolution at 0.143 FSC (Å)	8.0	4.9	8.3	5.2	7.3	Null
EMDB accession codes	EMD-52060	EMD-52052	EMD-52059	EMD-52051	EMD-47842	Null

Table S2. HIV-1 asymmetric flat lipid bilayer lipid composition. For each lipid species present in the membrane (specified by their CHARMM topology name), we list the total number of molecules in the membrane as well as the molar fraction and the fraction of molecules present in the intravirion and extravirion leaflet. The intravirion leaflet is rich in PIPs, phosphatidylethalonamine (PE) and phosphatidylserine (PS) while the outer leaflet is enriched in sphingomyelins (SM), ceramides (CER) , glycerides (GL) and phosphatidylcholine (PC). The intravirion and extravirion leaflets contain 1797 and 1964 lipid molecules, respectively, for a total of 3761 lipids in the bilayer membrane.

**Lipid (CHARMM)**	**No.Molecules (Total)**	**Mol.fraction (%)**	**Intravirio ratio**	**Extravirion ratio**	**No.Molecules (Intravirion)**	**No.Molecules (Extravirion)**
CER160	8	0.21	0.12	0.88	1	7
CER180	3	0.08	0.12	0.88	1	2
CHL1	1260	33.50	0.47	0.53	556	704
DLPC	83	2.21	0.2	0.8	15	68
DOPC	48	1.28	0.2	0.8	8	40
DPPC	109	2.90	0.28	0.72	25	84
NSM	129	3.43	0.12	0.88	10	119
PAPE	214	5.69	0.75	0.25	155	59
PAPS	78	2.07	0.81	0.19	62	16
PDOPC	15	0.40	0.18	0.82	3	12
PDOPE	217	5.77	0.92	0.08	197	20
PEPE	27	0.72	0.9	0.1	24	3
PEPS	127	3.38	0.75	0.25	93	34
PLPE	140	3.72	0.85	0.15	117	23
PLPS	84	2.23	0.76	0.24	62	22
POPC	141	3.75	0.16	0.84	19	122
POPE	20	0.53	0.75	0.25	15	5
POPS	26	0.69	0.74	0.26	18	8
SDPC	9	0.24	0.2	0.8	1	8
SDPE	146	3.88	0.88	0.12	126	20
SDPS	190	5.05	0.73	0.27	134	56
SSM	529	14.07	0.12	0.88	38	491
SAPI	42	1.12	0.85	0.15	35	7
SAPI14	19	0.51	0.85	0.15	16	3
SAPI24	68	1.81	0.85	0.15	57	11
SAPI34	1	0.03	0.85	0.15	1	0
POGL	28	0.74	0.34	0.66	8	20
Total	3761				1797	1964

Table S3. Lipid membrane and membrane in complex with MA systems built for MD simulations. The symmetric lipid bilayers (#1,#2) which were prepared using CHARMM-GUI [76] and equilibrated shortly before using the lipid coordinates as templates to build the asymmetric lipid membrane (#3). The apo HIV-1 asymmetric lipid membrane and in complex with the immature MA lattices (WT, L20K and L20K/E73K/A82T) were simulated for 1 µs (#4-#6) before calculating lateral lipid displacement, lipid headgroup occupancy and protein-lipid contact occupancies. Mature MA lattice perturbation simulations (#7-#13) via adsorption to the lipid membrane were performed for 60 ns varying the Langevincoupling constant from 10 ps-1 to 5ps-1 and 1 ps-1 over periods of 20 ns allowing the MA and membrane to progressively come together and probing the MA-MA intertrimer and intratrimer interactions and assess the MA lattice structural stability. All simulations were performed on NPT ensembles with constant ratio control.

Simulation #	Description	Dimensions (Å)	# atoms	# wat. Mol.	Simulation length (ns)	Number of replicas
1	HIV-1 intravirion symmetric lipid membrane	317.8 x 317.8 x 86.7	905,725	146,825	50	1
2	HIV-1 extravirion symmetric lipid membrane	293.6 x 293.6 x 91.2	831,950	131,146	50	1
3	HIV-1 asymmetric lipid membrane	294.2 x 294.2 x 90.5	819,471	131,634	1000	3
4	Immature WT MA lattice in complex w/ lipid membrane	292.9 x 292.9 x 153.5	1,367,306	289,132	1000	3
5	Immature L20K MA lattice in complex w/ lipid membrane	292.8 x 292.8 x 153.7	1,367,345	289,093	1000	3
6	Immature L20K/E73K/A82T MA lattice in complex w/ lipid membrane	293.3 x 293.3 x 153.2	1,367,618	289,015	1000	3
7	Mature WT MA lattice in complex w/ lipid membrane	293.9 x 293.9 x 151.9	1,363,130	287,740	60	6
8	Mature L20K MA lattice in complex w/ lipid membrane	294.0 x 294.0 x 151.7	1,363,169	287,701	60	6
9	Mature L20K/E73K/A82T MA lattice in complex w/ lipid membrane	293.5 x 293.5 x 152.4	1,362,950	287,537	60	6
10	Mature R19A MA lattice in complex w/ lipid membrane	294.1 x 294.1 x 151.4	1,362,506	287,701	60	6
11	Mature R19L MA lattice in complex w/ lipid membrane	294.1 x 294.1 x 151.4	1,362,857	287,701	60	6
12	Mature E41A MA lattice in complex w/ lipid membrane	294.1 x 294.1 x 151.4	1,362,857	287,701	60	6
13	Mature E51A MA lattice in complex w/ lipid membrane	294.1 x 294.1 x 151.4	1,362,857	287,701	60	6

Table S4 | Cryo-ET data acquisition and image processing for L20K

MA	Immature L20K	Mature L20K
**Data acquisition**		
Microscope	FEI Titan Krios	FEI Titan Krios
Energy-filter (eV)	20	20
Detector	Falcon4	Falcon4
Pixel size (A)	1.50	1.50
Defocus range (microns)	-1.5 to -5.5	-1.5 to -5.5
Acquisition scheme	-60°/60°, 3°, dose symmetric	-60°/60°, 3°, dose symmetric
Total Dose (electrons/A2)	123	~123
Frame number	10	10
Tomogram number (processed/total collected)	16	11
**Image processing**		
Virus particles	123	97
Wild type MA lattice template matching analysis	Yes	Yes

REFERENCES AND NOTES

1. S. A. Datta, F. Heinrich, S. Raghunandan, S. Krueger, J. E. Curtis, A. Rein, H. Nanda, HIV-1 Gag extension: Conformational changes require simultaneous interaction with membrane and nucleic acid. *J. Mol. Biol.* **406**, 205–214 (2011).

2. C. Aiken, P. Zhang, in *Advances in HIV-1 Assembly and Release* (Springer, 2013), pp. 153–166.

3. A. B. Kleinpeter, E. O. Freed, HIV-1 maturation: Lessons learned from inhibitors. *Viruses* **12**, 940 (2020).

4. W. I. Sundquist, H. G. Krausslich, HIV-1 assembly, budding, and maturation. *Cold Spring Harb. Perspect. Med.* **2**, a006924 (2012).

5. S. A. Datta, J. E. Curtis, W. Ratcliff, P. K. Clark, R. M. Crist, J. Lebowitz, S. Krueger, A. Rein, Conformation of the HIV-1 Gag protein in solution. *J. Mol. Biol.* **365**, 812–824 (2007).

6. S. A. Datta, Z. Zhao, P. K. Clark, S. Tarasov, J. N. Alexandratos, S. J. Campbell, M. Kvaratskhelia, J. Lebowitz, A. Rein, Interactions between HIV-1 Gag molecules in solution: An inositol phosphate-mediated switch. *J. Mol. Biol.* **365**, 799–811 (2007).

7. M. Bryant, L. Ratner, Myristoylation-dependent replication and assembly of human immunodeficiency virus 1. *Proc. Natl. Acad. Sci. U.S.A.* **87**, 523–527 (1990).

8. W. Zhou, L. J. Parent, J. W. Wills, M. D. Resh, Identification of a membrane-binding domain within the amino-terminal region of human immunodeficiency virus type 1 Gag protein which interacts with acidic phospholipids. *J. Virol.* **68**, 2556–2569 (1994).

9. C. Tang, E. Loeliger, P. Luncsford, I. Kinde, D. Beckett, M. F. Summers, Entropic switch regulates myristate exposure in the HIV-1 matrix protein. *Proc. Natl. Acad. Sci. U.S.A.* **101**, 517–522 (2004).

10. A. Ono, S. D. Ablan, S. J. Lockett, K. Nagashima, E. O. Freed, Phosphatidylinositol (4,5) bisphosphate regulates HIV-1 Gag targeting to the plasma membrane. *Proc. Natl. Acad. Sci. U.S.A.* **101**, 14889–14894 (2004).

11. V. Chukkapalli, I. B. Hogue, V. Boyko, W.-S. Hu, A. Ono, Interaction between the human immunodeficiency virus type 1 Gag matrix domain and phosphatidylinositol-(4, 5)-bisphosphate is essential for efficient gag membrane binding. *J. Virol.* **82**, 2405–2417 (2008).

12. F. Mucksch, V. Laketa, B. Muller, C. Schultz, H. G. Krausslich, Synchronized HIV assembly by tunable PIP_2 changes reveals PIP_2 requirement for stable Gag anchoring. *eLife* **6**, e25287 (2017).

13. J. S. Saad, J. Miller, J. Tai, A. Kim, R. H. Ghanam, M. F. Summers, Structural basis for targeting HIV-1 Gag proteins to the plasma membrane for virus assembly. *Proc. Natl. Acad. Sci. U.S.A.* **103**, 11364–11369 (2006).

14. J. Vlach, J. S. Saad, Trio engagement via plasma membrane phospholipids and the myristoyl moiety governs HIV-1 matrix binding to bilayers. *Proc. Natl. Acad. Sci. U.S.A.* **110**, 3525–3530 (2013).

15. C. P. Hill, D. Worthylake, D. P. Bancroft, A. M. Christensen, W. I. Sundquist, Crystal structures of the trimeric human immunodeficiency virus type 1 matrix protein: Implications for membrane association and assembly. *Proc. Natl. Acad. Sci. U.S.A.* **93**, 3099–3104 (1996).

16. A. Alfadhli, D. Huseby, E. Kapit, D. Colman, E. Barklis, Human immunodeficiency virus type 1 matrix protein assembles on membranes as a hexamer. *J. Virol.* **81**, 1472–1478 (2007).

17. A. Alfadhli, R. L. Barklis, E. Barklis, HIV-1 matrix organizes as a hexamer of trimers on membranes containing phosphatidylinositol-(4, 5)-bisphosphate. *Virology* **387**, 466–472 (2009).

18. K. Qu, Z. Ke, V. Zila, M. Anders-Osswein, B. Glass, F. Mucksch, R. Muller, C. Schultz, B. Muller, H. G. Krausslich, J. A. G. Briggs, Maturation of the matrix and viral membrane of HIV-1. *Science* **373**, 700–704 (2021).

19. X. Yu, X. Yuan, M. F. McLane, T. H. Lee, M. Essex, Mutations in the cytoplasmic domain of human immunodeficiency virus type 1 transmembrane protein impair the incorporation of Env proteins into mature virions. *J. Virol.* **67**, 213–221 (1993).

20. T. Dorfman, F. Mammano, W. A. Haseltine, H. G. Gottlinger, Role of the matrix protein in the virion association of the human immunodeficiency virus type 1 envelope glycoprotein. *J. Virol.* **68**, 1689–1696 (1994).

21. E. O. Freed, M. A. Martin, Virion incorporation of envelope glycoproteins with long but not short cytoplasmic tails is blocked by specific, single amino acid substitutions in the human immunodeficiency virus type 1 matrix. *J. Virol.* **69**, 1984–1989 (1995).

22. E. O. Freed, M. A. Martin, Domains of the human immunodeficiency virus type 1 matrix and gp41 cytoplasmic tail required for envelope incorporation into virions. *J. Virol.* **70**, 341–351 (1996).

23. P. R. Tedbury, S. D. Ablan, E. O. Freed, Global rescue of defects in HIV-1 envelope glycoprotein incorporation: Implications for matrix structure. *PLOS Pathog.* **9**, e1003739 (2013).

24. P. R. Tedbury, M. Novikova, S. D. Ablan, E. O. Freed, Biochemical evidence of a role for matrix trimerization in HIV-1 envelope glycoprotein incorporation. *Proc. Natl. Acad. Sci. U.S.A.* **113**, E182–E190 (2016).

25. P. R. Tedbury, M. Novikova, A. Alfadhli, Y. Hikichi, I. Kagiampakis, V. N. KewalRamani, E. Barklis, E. O. Freed, HIV-1 matrix trimerization-impaired mutants are rescued by matrix substitutions that enhance envelope glycoprotein incorporation. *J. Virol.* **94**, e01526-19 (2019).

26. R. E. Murphy, J. S. Saad, The interplay between HIV-1 Gag binding to the plasma membrane and Env incorporation. *Viruses* **12**, 548 (2020).

27. F. Mammano, E. Kondo, J. Sodroski, A. Bukovsky, H. Göttlinger, Rescue of human immunodeficiency virus type 1 matrix protein mutants by envelope glycoproteins with short cytoplasmic domains. *J. Virol.* **69**, 3824–3830 (1995).

28. H. Reil, A. A. Bukovsky, H. R. Gelderblom, H. G. Göttlinger, Efficient HIV-1 replication can occur in the absence of the viral matrix protein. *EMBO J.* **17**, 2699–2708 (1998).

29. D. J. Wyma, J. Jiang, J. Shi, J. Zhou, J. E. Lineberger, M. D. Miller, C. Aiken, Coupling of human immunodeficiency virus type 1 fusion to virion maturation: A novel role of the gp41 cytoplasmic tail. *J. Virol.* **78**, 3429–3435 (2004).

30. T. Murakami, S. Ablan, E. O. Freed, Y. Tanaka, Regulation of human immunodeficiency virus type 1 Env-mediated membrane fusion by viral protease activity. *J. Virol.* **78**, 1026–1031 (2004).

31. J. Chojnacki, T. Staudt, B. Glass, P. Bingen, J. Engelhardt, M. Anders, J. Schneider, B. Muller, S. W. Hell, H. G. Krausslich, Maturation-dependent HIV-1 surface protein redistribution revealed by fluorescence nanoscopy. *Science* **338**, 524–528 (2012).

32. R. E. Kiernan, A. Ono, E. O. Freed, Reversion of a human immunodeficiency virus type 1 matrix mutation affecting Gag membrane binding, endogenous reverse transcriptase activity, and virus infectivity. *J. Virol.* **73**, 4728–4737 (1999).

33. C. Sumner, A. Ono, The “basics” of HIV-1 assembly. *PLOS Pathog.* **20**, e1011937 (2024).

34. R. E. Kiernan, A. Ono, G. Englund, E. O. Freed, Role of matrix in an early postentry step in the human immunodeficiency virus type 1 life cycle. *J. Virol.* **72**, 4116–4126 (1998).

35. R. E. Kiernan, E. O. Freed, Cleavage of the murine leukemia virus transmembrane env protein by human immunodeficiency virus type 1 protease: Transdominant inhibition by matrix mutations. *J. Virol.* **72**, 9621–9627 (1998).

36. Y. Hikichi, E. Takeda, M. Fujino, E. Nakayama, T. Matano, T. Murakami, HIV-1 matrix mutations that alter gag membrane binding modulate mature core formation and post-entry events. *Virology* **532**, 97–107 (2019).

37. C. Aiken, Cell-free assays for HIV-1 uncoating. *Methods Mol. Biol.* **485**, 41–53 (2009).

38. J. Zivanov, J. Oton, Z. Ke, A. von Kugelgen, E. Pyle, K. Qu, D. Morado, D. Castano-Diez, G. Zanetti, T. A. M. Bharat, J. A. G. Briggs, S. H. W. Scheres, A Bayesian approach to single-particle electron cryo-tomography in RELION-4.0. *eLife* **11**, e83724 (2022).

39. B. A. Himes, P. Zhang, emClarity: Software for high-resolution cryo-electron tomography and subtomogram averaging. *Nat. Methods* **15**, 955–961 (2018).

40. A. B. Samal, T. J. Green, J. S. Saad, Atomic view of the HIV-1 matrix lattice; implications on virus assembly and envelope incorporation. *Proc. Natl. Acad. Sci. U.S.A.* **119**, e2200794119 (2022).

41. M. Chavent, T. Reddy, J. Goose, A. C. E. Dahl, J. E. Stone, B. Jobard, M. S. Sansom, Methodologies for the analysis of instantaneous lipid diffusion in md simulations of large membrane systems. *Faraday Discuss.* **169**, 455–475 (2014).

42. S. Mattei, A. Tan, B. Glass, B. Muller, H. G. Krausslich, J. A. G. Briggs, High-resolution structures of HIV-1 Gag cleavage mutants determine structural switch for virus maturation. *Proc. Natl. Acad. Sci. U.S.A.* **115**, E9401–E9410 (2018).

43. R. Connor, F. A. Cardillo, R. Moss, F. Rabitti, in *Similarity Search and Applications: 6th International Conference, SISAP 2013, A Coruña, Spain, October 2-4, 2013, Proceedings 6* (Springer, 2013), pp. 163–168.

44. J. Lin, Divergence measures based on the Shannon entropy. *IEEE Trans. Inf. Theory* **37**, 145–151 (1991).

45. B. R. Miller III, T. D. McGee Jr., J. M. Swails, N. Homeyer, H. Gohlke, A. E. Roitberg, MMPBSA. py: An efficient program for end-state free energy calculations. *J. Chem. Theory Comput.* **8**, 3314–3321 (2012).

46. S. Genheden, U. Ryde, The MM/PBSA and MM/GBSA methods to estimate ligand-binding affinities. *Expert Opin. Drug Discovery* **10**, 449–461 (2015).

47. T. Siebenmorgen, M. Zacharias, Computational prediction of protein–protein binding affinities. *Wiley Interdiscip. Rev. Comput. Mol. Sci.* **10**, e1448 (2020).

48. E. O. Freed, G. Englund, M. A. Martin, Role of the basic domain of human immunodeficiency virus type 1 matrix in macrophage infection. *J. Virol.* **69**, 3949–3954 (1995).

49. C. Sumner, A. Ono, Relationship between HIV-1 Gag multimerization and membrane binding. *Viruses* **14**, 622 (2022).

50. L. S. Ehrlich, S. Fong, S. Scarlata, G. Zybarth, C. Carter, Partitioning of HIV-1 Gag and Gag-related proteins to membranes. *Biochemistry* **35**, 3933–3943 (1996).

51. J. C. V. Stacey, D. Hrebik, E. Nand, S. D. Shetty, K. Qu, M. Boicu, M. Anders-Osswein, P. D. Uchil, R. A. Dick, W. Mothes, H. G. Krausslich, B. Muller, J. A. G. Briggs, The conserved HIV-1 spacer peptide 2 triggers matrix lattice maturation. *Nature* **640**, 258–264 (2025).

52. L. V. Coren, J. A. Thomas, E. Chertova, R. C. Sowder II, T. D. Gagliardi, R. J. Gorelick, D. E. Ott, Mutational analysis of the C-terminal gag cleavage sites in human immunodeficiency virus type 1. *J. Virol.* **81**, 10047–10054 (2007).

53. A. de Marco, A. M. Heuser, B. Glass, H. G. Krausslich, B. Muller, J. A. Briggs, Role of the SP2 domain and its proteolytic cleavage in HIV-1 structural maturation and infectivity. *J. Virol.* **86**, 13708–13716 (2012).

54. B. Muller, M. Anders, H. Akiyama, S. Welsch, B. Glass, K. Nikovics, F. Clavel, H. M. Tervo, O. T. Keppler, H. G. Krausslich, HIV-1 Gag processing intermediates trans-dominantly interfere with HIV-1 infectivity. *J. Biol. Chem.* **284**, 29692–29703 (2009).

55. A. Adachi, H. E. Gendelman, S. Koenig, T. Folks, R. Willey, A. Rabson, M. A. Martin, Production of acquired immunodeficiency syndrome-associated retrovirus in human and nonhuman cells transfected with an infectious molecular clone. *J. Virol.* **59**, 284–291 (1986).

56. A. Engelman, G. Englund, J. M. Orenstein, M. A. Martin, R. Craigie, Multiple effects of mutations in human immunodeficiency virus type 1 integrase on viral replication. *J. Virol.* **69**, 2729–2736 (1995).

57. A. Ono, E. O. Freed, Plasma membrane rafts play a critical role in HIV-1 assembly and release. *Proc. Natl. Acad. Sci. U.S.A.* **98**, 13925–13930 (2001).

58. T. Murakami, E. O. Freed, The long cytoplasmic tail of gp41 is required in a cell type-dependent manner for HIV-1 envelope glycoprotein incorporation into virions. *Proc. Natl. Acad. Sci.* **97**, 343–348 (2000).

59. V. Varthakavi, P. J. Browning, P. Spearman, Human immunodeficiency virus replication in a primary effusion lymphoma cell line stimulates lytic-phase replication of Kaposi's sarcoma-associated herpesvirus. *J. Virol.* **73**, 10329–10338 (1999).

60. J. Sodroski, W. C. Goh, C. Rosen, A. Dayton, E. Terwilliger, W. Haseltine, A second post-transcriptional trans-activator gene required for HTLV-III replication. *Nature* **321**, 412–417 (1986).

61. Y. Hikichi, J. R. Grover, A. Schäfer, W. Mothes, E. O. Freed, Epistatic pathways can drive HIV-1 escape from integrase strand transfer inhibitors. *Sci. Adv.* **10**, eadn0042 (2024).

62. Y. Hikichi, R. Van Duyne, P. Pham, J. L. Groebner, A. Wiegand, J. W. Mellors, M. F. Kearney, E. O. Freed, Mechanistic analysis of the broad antiretroviral resistance conferred by HIV-1 envelope glycoprotein mutations. *mBio* **12**, e03134-20 (2021).

63. C. Akil, R. C. Robinson, Genomes of Asgard archaea encode profilins that regulate actin. *Nature* **562**, 439–443 (2018).

64. S. Q. Zheng, E. Palovcak, J. P. Armache, K. A. Verba, Y. Cheng, D. A. Agard, MotionCor2: Anisotropic correction of beam-induced motion for improved cryo-electron microscopy. *Nat. Methods* **14**, 331–332 (2017).

65. J. R. Kremer, D. N. Mastronarde, J. R. McIntosh, Computer visualization of three-dimensional image data using IMOD. *J. Struct. Biol.* **116**, 71–76 (1996).

66. T. Ni, T. Frosio, L. Mendonca, Y. Sheng, D. Clare, B. A. Himes, P. Zhang, High-resolution in situ structure determination by cryo-electron tomography and subtomogram averaging using emClarity. *Nat. Protoc.* **17**, 421–444 (2022).

67. J. Ning, G. Erdemci-Tandogan, E. L. Yufenyuy, J. Wagner, B. A. Himes, G. Zhao, C. Aiken, R. Zandi, P. Zhang, In vitro protease cleavage and computer simulations reveal the HIV-1 capsid maturation pathway. *Nat. Commun.* **7**, 13689 (2016).

68. U. H. Ermel, S. M. Arghittu, A. S. Frangakis, ArtiaX: An electron tomography toolbox for the interactive handling of sub-tomograms in UCSF ChimeraX. *Protein Sci.* **31**, e4472 (2022).

69. T. D. Goddard, C. C. Huang, E. C. Meng, E. F. Pettersen, G. S. Couch, J. H. Morris, T. E. Ferrin, UCSF ChimeraX: Meeting modern challenges in visualization and analysis. *Protein Sci.* **27**, 14–25 (2018).

70. M. Beckers, A. J. Jakobi, C. Sachse, Thresholding of cryo-EM density maps by false discovery rate control. *IUCrJ* **6**, 18–33 (2019).

71. F. Mucksch, M. Citir, C. Luchtenborg, B. Glass, A. Traynor-Kaplan, C. Schultz, B. Brugger, H. G. Krausslich, Quantification of phosphoinositides reveals strong enrichment of PIP_2 in HIV-1 compared to producer cell membranes. *Sci. Rep.* **9**, 17661 (2019).

72. A. J. Bryer, T. Reddy, E. Lyman, J. R. Perilla, Full scale structural, mechanical and dynamical properties of HIV-1 liposomes. *PLOS Comput. Biol.* **18**, e1009781 (2022).

73. S. Park, W. Im, R. W. Pastor, Developing initial conditions for simulations of asymmetric membranes: A practical recommendation. *Biophys. J.* **120**, 5041–5059 (2021).

74. S. Jo, T. Kim, V. G. Iyer, W. Im, CHARMM-GUI: A web-based graphical user interface for CHARMM. *J. Comput. Chem.* **29**, 1859–1865 (2008).

75. J. Lee, X. Cheng, J. M. Swails, M. S. Yeom, P. K. Eastman, J. A. Lemkul, S. Wei, J. Buckner, J. C. Jeong, Y. Qi, S. Jo, V. S. Pande, D. A. Case, C. L. Brooks III, A. D. MacKerell Jr., J. B. Klauda, W. Im, CHARMM-GUI input generator for NAMD, GROMACS, AMBER,

OpenMM, and CHARMM/OpenMM simulations using the CHARMM36 additive force field. *J. Chem. Theory Comput.* **12**, 405–413 (2016).

76. E. L. Wu, X. Cheng, S. Jo, H. Rui, K. C. Song, E. M. Dávila-Contreras, Y. Qi, J. Lee, V. Monje-Galvan, R. M. Venable, J. B. Klauda, W. Im, CHARMM-GUI *Membrane Builder* toward realistic biological membrane simulations. *J. Comput. Chem.* **35**, 1997–2004 (2014).

77. J. Lee, D. S. Patel, J. Stahle, S. J. Park, N. R. Kern, S. Kim, J. Lee, X. Cheng, M. A. Valvano, O. Holst, Y. A. Knirel, Y. Qi, S. Jo, J. B. Klauda, G. Widmalm, W. Im, CHARMM-GUI *Membrane Builder* for complex biological membrane simulations with glycolipids and lipoglycans. *J. Chem. Theory Comput.* **15**, 775–786 (2019).

78. Y. Gao, J. Lee, I. P. S. Smith, H. Lee, S. Kim, Y. Qi, J. B. Klauda, G. Widmalm, S. Khalid, W. Im, CHARMM-GUI supports hydrogen mass repartitioning and different protonation states of phosphates in lipopolysaccharides. *J. Chem. Inf. Model.* **61**, 831–839 (2021).

79. U. Essmann, L. Perera, M. L. Berkowitz, T. Darden, H. Lee, L. G. Pedersen, A smooth particle mesh Ewald method. *J. Chem. Phys.* **103**, 8577–8593 (1995).

80. J. C. Phillips, D. J. Hardy, J. D. C. Maia, J. E. Stone, J. V. Ribeiro, R. C. Bernardi, R. Buch, G. Fiorin, J. Henin, W. Jiang, R. McGreevy, M. C. R. Melo, B. K. Radak, R. D. Skeel, A. Singharoy, Y. Wang, B. Roux, A. Aksimentiev, Z. Luthey-Schulten, L. V. Kale, K. Schulten, C. Chipot, E. Tajkhorshid, Scalable molecular dynamics on CPU and GPU architectures with NAMD. *J. Chem. Phys.* **153**, 044130 (2020).

81. C. R. Søndergaard, M. H. Olsson, M. Rostkowski, J. H. Jensen, Improved treatment of ligands and coupling effects in empirical calculation and rationalization of p K a values. *J. Chem. Theory Comput.* **7**, 2284–2295 (2011).

82. M. H. Olsson, C. R. Søndergaard, M. Rostkowski, J. H. Jensen, PROPKA3: Consistent treatment of internal and surface residues in empirical p K a predictions. *J. Chem. Theory Comput.* **7**, 525–537 (2011).

83. W. Humphrey, A. Dalke, K. Schulten, VMD: Visual molecular dynamics. *J. Mol. Graph.* **14**, 33–38 (1996).

84. L. G. Trabuco, E. Villa, E. Schreiner, C. B. Harrison, K. Schulten, Molecular dynamics flexible fitting: A practical guide to combine cryo-electron microscopy and X-ray crystallography. *Methods* **49**, 174–180 (2009).

85. T. Giorgino, Computing diffusion coefficients in macromolecular simulations: The Diffusion Coefficient Tool for VMD. *J. Open Source Softw.* **4**, 1698 (2019).

86. A. J. Bryer, J. S. Rey, C. Xu, J. A. Hadden-Perilla, J. R. Perilla, "Unsupervised refinement of protein structures" in *Integrated Structural Biology* (Royal Society of Chemistry, 2023), pp. 327–347.

87. S. J. Fleishman, A. Leaver-Fay, J. E. Corn, E.-M. Strauch, S. D. Khare, N. Koga, J. Ashworth, P. Murphy, F. Richter, G. Lemmon, J. Meiler, D. Baker, RosettaScripts: A scripting language interface to the Rosetta macromolecular modeling suite. *PLOS ONE* **6**, e20161 (2011).

88. L. M. Amzel, X. Siebert, A. Armstrong, G. Pabon, Thermodynamic calculations in biological systems. *Biophys. Chem.* **117**, 239–254 (2005).

89. G. Pabón, L. M. Amzel, Mechanism of titin unfolding by force: Insight from quasi-equilibrium molecular dynamics calculations. *Biophys. J.* **91**, 467–472 (2006).

90. M. J. Abraham, T. Murtola, R. Schulz, S. Páll, J. C. Smith, B. Hess, E. Lindahl, GROMACS: High performance molecular simulations through multi-level parallelism from laptops to supercomputers. *SoftwareX* **1-2**, 19–25 (2015).

91. A. P. Nadkarni, *The Tcl Programming Language: A Comprehensive Guide* (CreateSpace Independent Publishing Platform, 2017).

92. P. Langevin, On the theory of brownian motion. *C. R. Acad. Sci.* **146**, 530 (1908).

93. J. C. Mattingly, A. M. Stuart, D. J. Higham, Ergodicity for SDEs and approximations: Locally Lipschitz vector fields and degenerate noise. *Stoch. Process. their Appl.* **101**, 185–232 (2002).

94. A. Kavalur, V. Guduguntla, W. K. Kim, Effects of Langevin friction and time steps in the molecular dynamics simulation of nanoindentation. *Mol. Simul.* **46**, 911–922 (2020).

95. J. Ruiz-Franco, L. Rovigatti, E. Zaccarelli, On the effect of the thermostat in non-equilibrium molecular dynamics simulations. *Eur. Phys. J.* **41**, 80 (2018).

96. A. Hinderliter, S. May, Cooperative adsorption of proteins onto lipid membranes. *J. Phys. Condens. Matter* **18**, S1257–S1270 (2006).

97. M. P. Muller, T. Jiang, C. Sun, M. Lihan, S. Pant, P. Mahinthichaichan, A. Trifan, E. Tajkhorshid, Characterization of lipid–protein interactions and lipid-mediated modulation of membrane protein function through molecular simulation. *Chem. Rev.* **119**, 6086–6161 (2019).

98. R. X. Ramirez, O. Campbell, A. J. Pradhan, G. E. Atilla-Gokcumen, V. Monje-Galvan, Modeling the molecular fingerprint of protein-lipid interactions of MLKL on complex bilayers. *Front. Chem.* **10**, 1088058 (2023).

99. R. A. Dick, V. M. Vogt, Membrane interaction of retroviral Gag proteins. *Front. Microbiol.* **5**, 187 (2014).

100. S. H. Ayed, A. D. Cloutier, L. J. McLeod, A. C. Foo, A. M. Damry, N. K. Goto, Dissecting the role of conformational change and membrane binding by the bacterial cell division regulator MinE in the stimulation of MinD ATPase activity. *J. Biol. Chem.* **292**, 20732–20743 (2017).

101. D. Sun, J. Forsman, C. E. Woodward, Amphipathic membrane-active peptides recognize and stabilize ruptured membrane pores: Exploring cause and effect with coarse-grained simulations. *Langmuir* **31**, 752–761 (2015).

102. R. A. Corey, P. J. Stansfeld, M. S. Sansom, The energetics of protein–lipid interactions as viewed by molecular simulations. *Biochem. Soc. Trans.* **48**, 25–37 (2020).

103. V. Monje-Galvan, G. A. Voth, Binding mechanism of the matrix domain of HIV-1 gag on lipid membranes. *eLife* **9**, e58621 (2020).

104. J. Vlach, J. S. Saad, Structural and molecular determinants of HIV-1 Gag binding to the plasma membrane. *Front. Microbiol.* **6**, 232 (2015).

105. M. S. Valdés-Tresanco, M. E. Valdés-Tresanco, P. A. Valiente, E. Moreno, gmx_MMPBSA: A new tool to perform end-state free energy calculations with GROMACS. *J. Chem. Theory Comput.* **17**, 6281–6291 (2021).

106. S. K. Panday, E. Alexov, Protein–protein binding free energy predictions with the MM/PBSA approach complemented with the gaussian-based method for entropy estimation. *ACS Omega* **7**, 11057–11067 (2022).

107. Y.-J. Sheng, Y.-W. Yin, Y.-Q. Ma, H.-M. Ding, Improving the performance of MM/PBSA in protein–protein interactions via the screening electrostatic energy. *J. Chem. Inf. Model.* **61**, 2454–2462 (2021).

108. D. A. Case, H. M. Aktulga, K. Belfon, I. Y. Ben-Shalom, J. T. Berryman, S. R. Brozell, D. S. Cerutti, T. E. Cheatham III, G. A. Cisneros, V. W. D. Cruzeiro, T. A. Darden, N. Forouzesh, M. Ghazimirsaeed, G. Giambaşu, T. Giese, M. K. Gilson, H. Gohlke, A. W. Goetz, J. Harris, Z. Huang, S. Izadi, S. A. Izmailov, K. Kasavajhala, M. C. Kaymak, A. Kovalenko, T. Kurtzman, T. S. Lee, P. Li, Z. Li, C. Lin, J. Liu, T. Luchko, R. Luo, M. Machado, M. Manathunga, K. M. Merz, Y. Miao, O. Mikhailovskii, G. Monard, H. Nguyen, K. A. O'Hearn, A. Onufriev, F. Pan, S. Pantano, A. Rahnamoun, D. R. Roe, A. Roitberg, C. Sagui, S. Schott-Verdugo, A. Shajan, J. Shen, C. L. Simmerling, N. R. Skrynnikov, J. Smith, J. Swails, R. C. Walker, J. Wang, J. Wang, X. Wu, Y. Wu, Y. Xiong, Y. Xue, D. M. York, C. Zhao, Q. Zhu, P. A. Kollman, *Amber 2022 Reference Manual (Covers Amber22 and AmberTools22)* (University of California, San Francisco, 2022).

109. M. Mirdita, K. Schütze, Y. Moriwaki, L. Heo, S. Ovchinnikov, M. Steinegger, ColabFold: Making protein folding accessible to all. *Nat. Methods* **19**, 679–682 (2022).

110. R. Evans, M. O'Neill, A. Pritzel, N. Antropova, A. Senior, T. Green, A. Žídek, R. Bates, S. Blackwell, J. Yim, Protein complex prediction with AlphaFold-Multimer. bioRxiv 463034 [Preprint] (2021). https://doi.org/10.1101/2021.10.04.463034.

111. F. K. Schur, M. Obr, W. J. Hagen, W. Wan, A. J. Jakobi, J. M. Kirkpatrick, C. Sachse, H. G. Krausslich, J. A. Briggs, An atomic model of HIV-1 capsid-SP1 reveals structures regulating assembly and maturation. *Science* **353**, 506–508 (2016).
